# A Comparison of Blood Plasma Small Extracellular Vesicle Enrichment Strategies for Proteomic Analysis

**DOI:** 10.3390/proteomes10020019

**Published:** 2022-06-01

**Authors:** Natalie P. Turner, Pevindu Abeysinghe, Keith A. Kwan Cheung, Kanchan Vaswani, Jayden Logan, Pawel Sadowski, Murray D. Mitchell

**Affiliations:** 1 Centre for Children’s Health Research (CCHR), Queensland University of Technology (QUT), 62 Graham St., South Brisbane, QLD 4101, Australia; abeysinghe.abeysingh@hdr.qut.edu.au (P.A.); k.kwancheung@hdr.qut.edu.au (K.A.K.C.); k.vaswani@uq.edu.au (K.V.); jayden.logan@qut.edu.au (J.L.); murray.mitchell@qut.edu.au (M.D.M.); 2 Faculty of Health, School of Biomedical Sciences, Kelvin Grove Campus, Queensland University of Technology (QUT), Victoria Park Rd., Kelvin Grove, QLD 4059, Australia; 3 Central Analytical Research Facility (CARF), Queensland University of Technology (QUT), 2 George St., Brisbane, QLD 4000, Australia; pawel.sadowski@qut.edu.au

**Keywords:** small extracellular vesicle, extracellular vesicle, exosome, isolation, enrichment, ultracentrifugation, size-exclusion chromatography, ultrafiltration, mass spectrometry, proteomics, extracellular vesicles

## Abstract

Proteomic analysis of small extracellular vesicles (sEVs) poses a significant challenge. A ‘gold-standard’ method for plasma sEV enrichment for downstream proteomic analysis is yet to be established. Methods were evaluated for their capacity to successfully isolate and enrich sEVs from plasma, minimise the presence of highly abundant plasma proteins, and result in the optimum representation of sEV proteins by liquid chromatography tandem mass spectrometry. Plasma from four cattle (Bos taurus) of similar physical attributes and genetics were used. Three methods of sEV enrichment were utilised: ultracentrifugation (UC), size-exclusion chromatography (SEC), and ultrafiltration (UF). These methods were combined to create four groups for methodological evaluation: UC + SEC, UC + SEC + UF, SEC + UC and SEC + UF. The UC + SEC method yielded the highest number of protein identifications (IDs). The SEC + UC method reduced plasma protein IDs compared to the other methods, but also resulted in the lowest number of protein IDs overall. The UC + SEC + UF method decreased sEV protein ID, particle number, mean and mode particle size, particle yield, and did not improve purity compared to the UC + SEC method. In this study, the UC + SEC method was the best method for sEV protein ID, purity, and overall particle yield. Our data suggest that the method and sequence of sEV enrichment strategy impacts protein ID, which may influence the outcome of biomarker discovery studies.

## 1. Introduction

The capability of instrumentation to analyse complex biological fluids and nanoparticles has advanced greatly in recent times. Advanced mass spectrometry (MS) and DNA sequencing platforms have become ubiquitous and accessible. Likewise, protocols for processing samples for downstream proteomics and genomics analyses have improved, which has had a positive impact on data quality and reproducibility [1,2]. A subpopulation of extracellular vesicles (EVs), small extracellular vesicles (sEVs), are nanoparticles of diameter < 200 nm and have been the focus of many studies relating to biomarker development and targeted therapeutics [3,4,5,6]. Small EVs are comprised of a range of EV subtypes, including exomeres (~35–50 nm), exosomes (EX; ~50–150 nm), microvesicles (~40–1000 nm) and apoptotic vesicles (~100–1000 nm) [7,8]. Typical EV cargo consists of nucleic acids, lipids and lipid-mediators, and proteins [3,9]. Thus, EVs and sEVs continue to hold global interest for their diagnostic and therapeutic potential [10,11,12,13,14]. However, their nano-size and complexity of cargo analysis continue to pose technical challenges. When derived from complex biological fluids such as plasma, multiple purification and enrichment steps are required, which inevitably reduce the overall particle yield [12,15]. Additionally, new evidence concerning the importance of EV subtypes has further complicated analyses of heterogenous or multivesicular samples [7,8]. These subtypes have considerable overlap with non-EV particles such as high-, low- and very low-density lipoproteins with regard to their size and/or density, which places further emphasis on the need for more than one enrichment method for optimal enrichment and depletion of interfering non-EV particles [9,16,17,18]. While there have been steady improvements in standardising enrichment protocols and improving particle purity and yield, there is as yet no consensus for the best method of enrichment. This is likely due to the multitude of biological fluids from which EVs and sEVs can be isolated and enriched, and the growing number of downstream applications now available to analyse the diverse EV cargos [19,20,21]. The use of multi-omics approaches in EV and sEV research is gaining popularity, giving way to a new wave of diagnostics. Recently, combined sEV biomarker panels have been developed that consist of a number of protein and miRNA candidates, and which have been shown to improve the sensitivity and specificity of pancreatic cancer screening [22].

Ultracentrifugation (UC) is one of the most widely used techniques for the enrichment of EVs from various types of biological fluids and cell culture media [23,24,25]. To increase purity of EV preparations, enrich for sEVs and decrease contaminant carryover, this is often followed or preceded by other enrichment or purification strategies, such as size-exclusion chromatography (SEC), or ultra-filtration (UF) [23,24,26,27]. SEC is commonly performed using commercially available columns, which separates nanoparticles based on their size using a resin with a variable pore size depending on the user’s requirements [28,29]. Particles larger than the pore size of the matrix used (e.g., 70 nm for Izon qEV original SEC columns) can be efficiently separated from other contaminants such as high-density lipoproteins [29]. However, as previously mentioned, other contaminating particles are within the EV size range, such as chylomicrons (100–600 nm) and very low-density lipoproteins (30–80 nm) [24]. UF is also performed using commercially available spin columns with a variable kDa cut-off filter, and is used to concentrate and/or filter particles [25]. The combination and order in which these enrichment processes are performed may influence the subpopulations of EVs obtained, the concentration, the particle sizes and/or purity of the final sample. It is unknown whether there is an optimal method and/or order of enrichment technique for specific downstream applications, such as MS-based proteomics [26,27,30,31]. In this study, we aim to determine the optimal enrichment method/s for proteomic analysis of sEVs isolated from plasma. Building on previous work from our group that employed a multi-step enrichment method to isolate sEVs from blood plasma, our approach utilised some of the most popular and widely accessible enrichment strategies to isolate and purify sEVs from biofluids; UC, SEC, and UF [20,23]. UC can be used to pellet EVs from larger sample volumes and therefore allows flexibility regarding the amount of starting material used. SEC is suitable for smaller volumes (500–1000 µL), so for this reason it is commonly used when sample volumes are limited. UF can be utilised with a range of starting volumes and has already been investigated in combination with SEC (UF-SEC) and compared to UC as a stand-alone enrichment strategy [32]. This study was performed using cell culture media and did not perform UF after SEC. A more recent study compared UC and SEC as standalone enrichment methods using blood plasma from Sprague-Dawley rats and found that although UC resulted in lower particle yields, it was the superior method for purity [33]. As these methods are so widely used and can be implemented and adjusted for a range of sample volumes, we aimed to determine whether performing UC before or after SEC would affect enrichment and/or purity for proteomics analysis. We then investigated whether applying UF after SEC as an alternative to UC, or as an additional purification step following UC and SEC in combination, would improve protein identification by MS. When referring to EV populations obtained from each method used in this study, the term ‘sEVs’ will be used to refer to all vesicles within the small EV size range (diameter < 200 nm), unless specifically stated otherwise.

As targets for biomarker discovery, sEVs have been under investigation in agricultural studies for their ability to predict health status and reproductive outcomes in dairy cows [34,35,36]. The knowledge of sEV enrichment efficiency may be beneficial in designing future biomarker discovery studies. Our group has experience working with bovine plasma, and we have therefore utilised samples from dairy cows for the purpose of this study.

## 2. Materials and Methods

### 2.1. Plasma Collection

Holstein-Friesian primiparous cows used in this study were part of a larger experiment performed by DairyNZ (Tokanui Farm, AgResearch), using an established model of dairy cow fertility as described by Meier et al. (2017) (approved by the Ruakura Animal Ethics Committee AEC#14200) [37]. The use of all samples in this study was also approved by the Queensland University of Technology Animal Ethics Committee (QV reference #83807 and #83810). Criteria for inclusion were if calving occurred between week 28 and 31 (inclusive) (week = year week) *n* = 96, which was between 9th July and 5th August 2017 (inclusive). Exclusion criteria were if a sample was missing (did not have both the milk and EDTA plasma samples), if there was no sample date recorded, or if the animal received a reproductive treatment. Additionally, cows were excluded if they had censored post-partum anoestrous interval (PPAI) data (i.e., if PPAI sampling ended before PPAI was confirmed). From this larger group (*n* = 80), stored blood plasma samples of dairy cows (*n* = 4) of similar physical attributes, fertility status and genetics were used in this study [38]. Animals were managed in a pasture-based, spring-calving dairy system. All experiments were performed in accordance with relevant guidelines and regulations. The authors confirm this study was carried out in compliance with ARRIVE guidelines (https://arriveguidelines.org/arrive-guidelines, accessed on 10 January 2022).

Blood samples for sEV enrichment were collected as previously described by Crookenden et al. (2016), with slight modification [35]. Briefly, blood was collected by coccygeal venepuncture into evacuated blood tubes containing lithium heparin anticoagulant. Blood was immediately placed on ice and centrifuged at 1500× *g* for 12 min at 4 °C. The plasma was aliquoted, frozen and stored at −80 °C until thawed for the following sEV isolation and enrichment methods. Twenty millilitres of plasma per replicate were thawed on ice the day sEV isolation and enrichment was initiated.

### 2.2. Small EV Isolation and Enrichment

#### 2.2.1. Pre-Treatment

**Figure 1:**
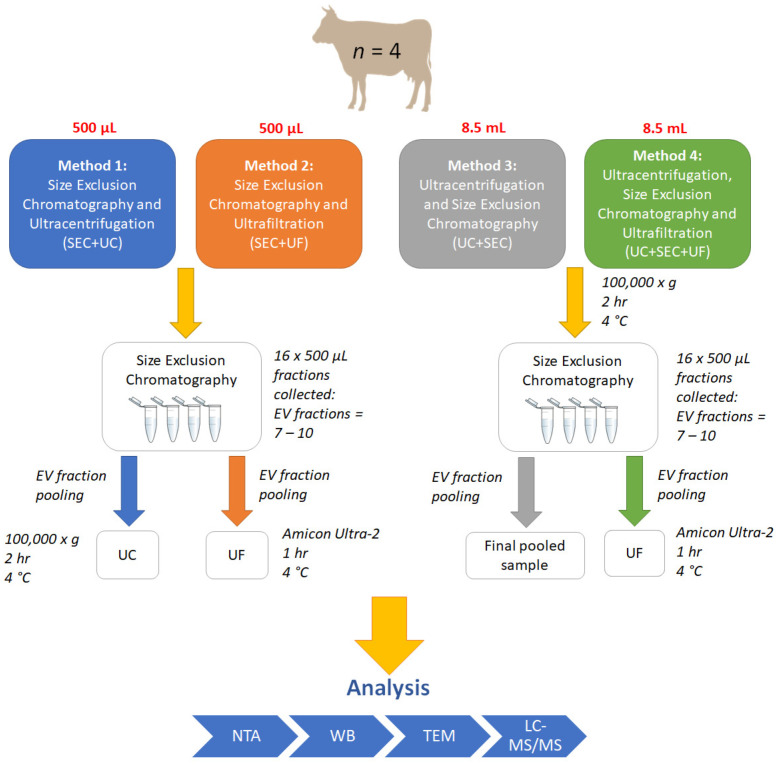
Workflow for small extracellular vesicle (sEV) enrichment methods 1–4. SEC: size-exclusion chromatography; UF: ultrafiltration; UC: ultracentrifugation; EV: extracellular vesicle; NTA: nanoparticle tracking analysis; WB: western blot; TEM: transmission electron microscopy; LC-MS/MS: liquid chromatography tandem mass spectrometry.

Plasma samples with a total volume of 20 mL were centrifuged at 3000× *g* for 10 min at 4 °C to remove debris, and the supernatant was collected. The supernatant was then centrifuged at 12,000× *g* for 30 min at 4 °C to remove apoptotic cell bodies. The supernatant was then collected and passed through a 0.22-µm filter (Corning Costar, Mulgrave, Australia), and two 500 µL aliquots from each biological sample were set aside and kept on ice for SEC. An 8.5 mL aliquot was then set aside for each UC method (see Figure 1 for workflow). An additional aliquot of plasma (250 µL) was also set aside for use as a non-sEV control in later proteomic analysis.

One column was used per two animals. In between uses, the columns were flushed with 0.5 mL 1M NaOH (Sigma-Aldrich (Merck), Melbourne, Australia) solution to clear debris, followed by 15–20 mL filtered Dulbecco’s phosphate buffered saline, pH 7.0–7.3 (DPBS).

#### 2.2.2. Size-Exclusion Chromatography (SEC)

Next, 500 µL of plasma from the previous step was passed through commercially available SEC columns, qEV original with matrix pore size 70 nm (Izon Science, Christchurch, New Zealand) as per manufacturer’s instructions, as previously described [39]. Briefly, the column was allowed to flow under gravitational force (1 drop every 1–2 s), and eluted drops collected into pre-labelled 1.5 mL microcentrifuge tubes. Tubes were changed every 500 µL of collection volume and placed immediately on ice for a total of 16 fractions.

#### 2.2.3. Ultrafiltration (UF)

Small EV-enriched fractions 7–10 were pooled by combining 400 µL of each to give a total volume of 1.6 mL. Pooled sEV-enriched samples were loaded onto pre-wetted Amicon Ultra-2 Centrifugal Filter Units with 3 kDa cut-off (UFC200324, Merck Millipore, Melbourne, Australia). Samples were concentrated as per manufacturer’s instructions for a total time of 80 min, to a final volume of ~300 µL. The concentrate was collected by reverse centrifugation as per manufacturer’s instructions. The concentrated samples were stored at −80 °C until further analysis.

#### 2.2.4. Method 1: SEC + UC

SEC was performed as described in previous SEC Section 2.2.2. Following SEC, pooled sEV samples of total volume 1.6 mL were transferred to 8.9 mL OptiSeal Polypropylene Tubes (361623, Beckman Coulter, Brea, CA, USA) and brought to equal volume with DPBS. Centrifugation of samples was performed at 100,000× *g* for 2 h at 4 °C (Type 70.1 Ti, Fixed angle ultracentrifuge rotor, Beckman Coulter, Brea, CA, USA). The supernatant was aspirated and the EV pellet was resuspended in 500 µL DPBS. Samples were stored at −80 °C until further analysis.

#### 2.2.5. Method 2: SEC + UF

SEC and UF were performed consecutively as described in the SEC and UF sections above (Section 2.2.2 and Section 2.2.3 respectively). The final concentrated sEV-enriched samples (fractions 7–10) were stored at −80 °C until further analysis.

#### 2.2.6. Method 3: UC + SEC

UC + SEC was performed as previously described [23]. The 500 µL EV samples were loaded onto SEC columns as described in the SEC section above (2.2.2). The sEV-enriched fractions 7–10 were pooled to give a final volume of 1.6 mL and 50 µL was aliquoted immediately for micro bicinchoninic acid (BCA) assay (see Section 2.4 below). The remaining samples were stored at 4 °C overnight prior to Western blot (WB) analysis.

#### 2.2.7. Method 4: UC + SEC + UF

Samples for UC + SEC + UF were subjected to the same methods as described in the UC + SEC section above (Section 2.2.6), followed by UF, as described in Section 2.2.3. The final concentrated volumes were ~100 µL. The concentrated samples were stored at 4 °C overnight prior to WB analysis.

### 2.3. Sample Pooling and Individual SEC Fraction Analysis

All methods underwent sample pooling by combining sEV SEC fractions 7, 8, 9 and 10 by equal volume (400 µL) to a total volume of 1.6 mL. A small volume of individual sEV-enriched fractions 7–10 (100 µL/each) was retained for nanoparticle tracking analysis (NTA) and WB.

In the case of individual fraction analysis resulting from SEC, this was performed after UC + SEC or after SEC. As the SEC fractions were pooled in order to perform SEC + UC, SEC + UF and UC + SEC + UF, individual fraction analysis after pooling was not possible.

### 2.4. Protein Quantification

Quantification of the total protein of the pooled and/or concentrated samples resulting from the described methods 1–4 was determined by micro BCA™ Protein Assay Kit (cat number 23235, Thermofisher Scientific, Brisbane, Australia) following the microplate assay protocol as per manufacturer’s instructions, as previously described [39]. Samples and bovine serum albumin (BSA) protein standards provided with the micro BCA assay kit were solubilised in 1% *w*/*v* sodium deoxycholate (SDC) (Sigma-Aldrich (Merck), Melbourne, Australia)/deionized H_2_O. Standards and samples were plated onto a 96-well flat-bottom plate (Greiner CELLSTAR®, Sigma-Aldrich (Merck), Melbourne, Australia), incubated for 2 hr at 37 °C, and absorbance read at 562 nm.

### 2.5. Western Blot

For visualisation of the residual BSA in the collected SEC fractions 1–16, equal volumes (10 µL) of sample from the pooled SEC fractions 1–6, and the individual SEC fractions 7–16, were aliquoted for WB analysis, as previously described [23]. Briefly, samples were transferred to an ice bath and sonicated for 2 min. Samples were then placed on ice and 4× NuPAGE LDS sample buffer (NP0007, Thermofisher Scientific, Brisbane, Australia) and 10× NuPAGE sample reducing agent (NP0004, Thermofisher Scientific, Brisbane, Australia) were added to give a final concentration of 1×, and reduced for 10 min at 70 °C, as per the manufacturer’s instructions. For visualisation of Flotillin-1 (FLOT-1) in pooled sEV-enriched (7–10) and non-sEV-enriched (11–16) fractions, the same procedure was followed as for BSA, with slight modification. An aliquot containing 2.5 µg of total protein (methods UC + SEC, UC + SEC + UF, SEC + UF) or 2 µg of total protein (SEC + UC) was mixed with an equal volume of 2% sodium dodecyl sulfate (SDS) (Sigma-Aldrich (Merck), Melbourne, Australia) in deionized H_2_O, heated at 95 °C for 3 min, and sonicated for 2 min. Samples were dried in a vacuum concentrator (cat number 5305000380, Eppendorf Concentrator plus, Sydney, Australia) and resuspended to a final concentration of 0.5 µg/µL, with 5 µL loaded per sample. Samples were resolved by electrophoresis on NuPAGE™ 4 to 12%, Bis-Tris, 1.0 mm, Mini Protein Gels, 15-well (NP0336BOX, Thermofisher Scientific, Brisbane, Australia) or 10-well (NP0321BOX, Thermofisher Scientific, Brisbane, Australia) with Chameleon^®^ Duo Pre-stained Protein Ladder (928-60000, Li-COR, Mulgrave, Australia). The protein gel was transferred onto a polyvinylidene fluoride membrane (Bio-Rad Laboratories Pty Ltd., Sydney, Australia) using the Trans-Blot Turbo system. Membranes were briefly washed in phosphate buffered saline containing 0.1% Tween-20 (PBST) (Sigma-Aldrich (Merck), Melbourne, Australia), before blocking in 5 mL Odyssey Intercept blocking buffer (927-70001, Li-COR, Mulgrave, Australia) and 5 mL phosphate buffered saline (PBS) (Sigma-Aldrich (Merck), Melbourne, Australia) for 1 hr at RT. The primary antibody was diluted with 1:1 Odyssey Blocking buffer, PBS, and Tween-20 added to final concentration of 0.1%. Samples were incubated with primary antibody overnight; anti-BSA (1:5000 dilution, Rabbit polyclonal (ab192603, Abcam, Melbourne, Australia); recombinant anti-Flotillin-1 (1:1000 dilution, Rabbit monoclonal (ab133497, Abcam, Melbourne, Australia) [40,41]. The next day, membranes were washed four times in PBST for 5 min each, and the membranes were incubated with secondary antibody for 1 hr at RT in the dark with gentle rocking; Goat anti-Rabbit IgG (1:15,000 dilution, Li-COR, Mulgrave, Australia). The secondary antibody was diluted with 1:1 Odyssey Intercept blocking buffer, PBS, and Tween-20 added to a final concentration of 0.1%. The membranes were washed in PBST four times for 5 min each. Membranes were rinsed briefly in PBS and imaged with Li-COR Odyssey fluorescent scanner at 700 and 800 nm. All images were processed using Image Studio Lite v5.2 (Li-COR Biosciences, Lincoln, NE, USA). Contrast and brightness were adjusted equally across entire images to best visualise protein bands.

### 2.6. Nanoparticle Tracking Analysis (NTA)

Measurements of particle size and concentration were performed using a NanoSight NS500 instrument (NanoSight NTA 3.1 Build 3.1.46, Malvern Panalytical, Sydney, Australia) as previously described [39]. Synthetic (latex) beads of size 100 nm were used to perform instrument calibration at a 1:250 dilution in deionized water. Measurements were performed on individual sEV fractions resulting from SEC. Pooled or concentrated samples resulting from the SEC + UF, UC + SEC + UF, SEC + UC and UC + SEC methods were analysed separately. NTA data were compiled into one .xls file before being transferred and analysed in GraphPad Prism (v9.1.2) (GraphPad, San Diego, CA, USA).

### 2.7. Statistical Analyses

NTA data were input and analysed using GraphPad Prism v9.1.2. Mean and mode size of pooled sEV samples resulting from each method under study were compared using a two-way analysis of variance (ANOVA) and multiple comparisons by isolation method, with significance set to *p* < 0.05. The same analysis was performed for particle concentration and protein concentration between all methods. Data were also assessed for normality of residuals and all data passed normality testing (alpha = 0.05).

### 2.8. Transmission Electron Microscopy (TEM)

Imaging of samples by TEM was outsourced and performed by the Central Analytical Research Facility microscopy laboratory, Queensland University of Technology (Brisbane, Australia). Pooled sEV-enriched samples for each biological replicate were imaged for each method of sEV isolation and enrichment. A representative biological replicate was chosen for individual fraction analysis for UC + SEC vs. SEC. Samples were drop mounted onto formvar coated 200 mesh Cu grids for 1 min, the excess was wicked away, and the mounted sample was negatively stained with 2% uranyl acetate (UA) (ProSciTech, Townsville, Australia) for 3 min. The excess UA was wicked away with filter paper and left to air dry. If dilution was required, samples were remounted as previously described, at a 50% dilution in deionized H_2_O. Samples were imaged on a JEOL JEM-1400 TEM (JEOL, Sydney, Australia) operated at 100 kV, mounted with a 2K TVIPS CCD camera (TVIPS, Gauting, Germany).

### 2.9. Protein Digestion

Pooled sEV-enriched samples for each biological replicate were combined for each method to create one master pool per method, and the master pools were processed for MS analysis using a modified filter aided sample preparation (FASP) method, as previously described [2,39]. Briefly, a volume of protein extract corresponding to 10–20 µg total protein was mixed 1:1 with 1% *w*/*v* SDC lysis buffer containing 100 mM dithiothreitol (DTT) (Sigma-Aldrich (Merck), Melbourne, Australia) and 100 mM Tris-HCL (pH 8.5) (Astral Scientific, Sydney, Australia). Samples were loaded onto a centrifugal device with 30 kDa molecular weight cut-off filter (30K Nanosep Centrifugal Device with Omega Membrane, PALL, Brisbane, Australia) and processed for LC-MS/MS by on-filter alkylation with 50 mM iodoacetamide (IAA) (Sigma-Aldrich (Merck), Melbourne, Australia) and 8M Urea (Sigma-Aldrich (Merck), Melbourne, Australia). Overnight enzymatic digestion was performed on-filter by adding trypsin (Trypsin Gold, Mass Spectrometry Grade, Promega, Sydney, Australia) at an enzyme to protein ratio of 1:50. Following overnight tryptic digestion, digested peptide samples were eluted into clean 1.5 mL microcentrifuge tubes prior to desalting.

### 2.10. Peptide Desalting

To acidify peptide digests prior to desalting, 4% trifluoroacetic acid (TFA) (Sigma-Aldrich (Merck), Melbourne, Australia) solution was added to eluted peptides at a 1:1 ratio. Acidified peptide digests were desalted using a double SCX membrane StageTip as previously described [39]. Peptides were dried in a vacuum centrifuge (SpeedVac, Eppendorf, Sydney, Australia) and reconstituted in 20 μL indexed retention time (iRT) buffer (Biognosys-11).

### 2.11. Peptide Assay

The peptide concentrations of resuspended peptide samples were determined using the Pierce™ Quantitative Colorimetric Peptide Assay (cat number 23275, Thermofisher Scientific, Brisbane, Australia) according to the manufacturer’s instructions. An appropriate addition of iRT buffer was used to equalize all peptide concentrations prior to analysis by LC-MS/MS.

### 2.12. Mass-Spectrometry (MS)

All peptide samples were analysed by LC-MS/MS as previously described [39]. Each sample contained 4 µg of digested peptides and in a 9 µL injection volume. Briefly, an Eksigent ekspert nanoLC 400 System (Eksigent Technologies, Redwood City, CA, USA) was used to perform reversed-phase chromatography using trapping for 3 min at a flow rate of 10 μL/min onto a Trajan ProteCol trap (120 Å, 3 μm, 10 mm × 300 μm, Trajan Scientific and Medical, Melbourne, Australia). Chromatographic separation was performed on an Eksigent ChromXP C18 3 μm 120 Å (3C18-CL-120, 3 μm, 120 Å, 0.3 × 150 mm, Eksigent Technologies, Redwood City, CA, USA) analytical column at a flow rate of 5 μL/min maintained at 40 °C. The mobile phase A consisted of 0.1% FA in water, and mobile phase B was made of 0.1% FA in ACN. A 68 min linear gradient of 3–25% mobile phase B followed by 5 min linear gradient of 25–35% mobile phase B was used to separate peptides. Following peptide elution, column was performed with 80% mobile phase B for 5 min and re-equilibrated with 97% mobile phase A for 8 min prior to the next injection. A triple time-of-flight (TOF) 6600 (SCIEX) instrument equipped with DuoSpray Ion Source configured for micro flow HPLC applications was used to conduct mass spectrometry.

### 2.13. Data-Dependent Acquisition-Mass Spetrometry (DDA-MS) Data Acquisition

Data acquisition for peptide samples was performed on the triple TOF 6600 as previously described [39]. TOF MS high resolution (30,000) scans were collected for 0.25 s over the *m*/*z* 400–1250 mass range. This was followed by TOF MS/MS high sensitivity scans on up to the 30 most abundant peptide ions over the range of *m*/*z* 100–1800 (0.05 s per each scan), which had intensities greater than 150 cps and a charge state of 2–5. The duration of dynamic exclusion was set at 15 s. Rolling collision energy with the collision energy spread set to 5 eV was used for ion fragmentation, and declustering potential was set to 80 V. The remaining gas and source parameters were adjusted as required.

### 2.14. Protein Identification

ProteinPilot (v. 5.0.2.0, 5346) (AB SCIEX LLC, Framingham, MA, USA) was used to process individual MS data files using Paragon Algorithm (v. 5.0.2.0, 5174) within the ProteinPilot software. Spectra were searched against a combined cattle proteome (23,847 sequences, downloaded Aug 2020, available in fasta format, Uniprot), cRAP sequences (ftp://ftp.thegpm.org/fasta/cRAP (accessed on 13 April 2021)) and iRT peptides file. Search parameters were entered as previously described [39]. The protein list was exported to a .xls file and subjected to an additional refinement where proteins for each method were filtered to include a minimum of two peptides per protein ID (1% FDR at the protein level; 5% FDR at the peptide level). All sEV samples were compared to a plasma control sample.

### 2.15. Gene Ontology Analysis

Gene ontology (PANTHERGO, Gene Ontology Phylogenetic Annotation Project, v 16.0. Available online: http://www.pantherdb.org (accessed on 30 April 2021)) was performed for the final list of proteins resulting from Section 2.14. Proteins were searched against species *Bos taurus* and separated by functional classification. FunRich (Functional Enrichment analysis tool, version 3.1.3, open-source software), March 2017, available at http://www.funrich.org/ (accessed on 10 June 2021)) was used to perform enrichment analysis. Proteins identified from each enrichment method were compared to the plasma control sample. The complete Vesiclepedia database (version 4.1, downloaded on 27 July 2021, available at http://microvesicles.org/Archive/VESICLEPEDIA_PROTEIN_MRNA_DETAILS_4.1.txt, accessed on 10 June 2021) was imported into FunRich, and all data were searched against the cattle proteome.

## 3. Results

### 3.1. Western Blot

**Figure 2:**
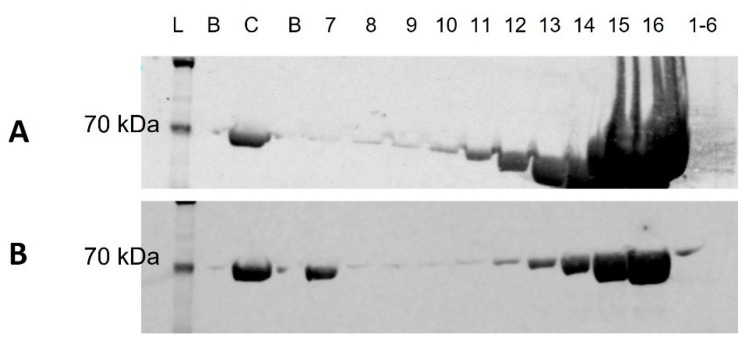
Western blot of bovine serum albumin (BSA) in SEC (**A**) and UC + SEC (**B**) fractions. L = ladder; C = Control, 1 ug purified BSA; B = blank; 7, 8, 9, 10, 11, 12, 13, 14, 15, 16, 1–6 = individual sEV (7–10), non-sEV (11–16) fractions, and void volume fractions pool (1–6). Predicted molecular weight of BSA = 69 kDa. (Full length blot images available in Supplementary File S2—Figures S1 and S2).

Serum albumin, and in the case of this study, BSA, is a highly abundant plasma protein. Optimal identification of sEV proteins depends upon efficient depletion of BSA from sEV enriched samples prior to MS analysis. WB analysis was performed on individual fractions resulting from UC + SEC and SEC to determine whether performing UC prior to SEC altered the relative abundance of BSA in the individual fractions, and if so, which fractions were affected. Individual fractions could not be assessed for methods ending with UC/UF, as sEV fractions were pooled to conduct these methods (SEC + UC, SEC + UF, UC + SEC + UF). However, all methods underwent UC + SEC or SEC prior to further processing and thus individual fraction analysis at this level is applicable to all four methods under study. Blots were probed with anti-BSA antibody to determine the BSA elution profile and the relative abundance of BSA in sEV fractions 7–10. SEC resulted in low BSA signal in sEV fractions 7–9, with an increase in intensity observed in sEV fraction 10, and a significant increase from non-sEV fraction 11 onwards (Figure 2A). In the case of UC + SEC, there was a stronger BSA signal in sEV fraction 7 as compared to sEV fractions 8–10, with signal increasing from non-sEV fraction 12 onwards (Figure 2B).

As exosomes are of particular interest in biomarker studies, the pooled samples resulting from each of the four methods were evaluated for the presence of a commonly identified exosome marker, FLOT-1. Additionally, the detection of FLOT-1 has been found to vary depending on the enrichment strategy and the biofluid being analysed [7,42]. As such, it may be of particular importance to researchers who wish to focus on one specific sEV subtype. FLOT-1 was not identified in any of the pooled sEV and non-sEV fractions (Supplementary File S2—Figures S3–S5). As human placental homogenates are positive for FLOT-1 expression, the human placental choriocarcinoma cell line JEG-3 was used as a positive control [43].

### 3.2. NTA

#### 3.2.1. Particle Concentration Profiles and Particle Yield

The particle concentrations obtained by SEC and UC + SEC in fractions 1–16 were compared using NTA to determine whether the addition of UC prior to SEC changed the elution profile of samples and if so, in which fractions these changes occurred. The overlaid profiles (Supplementary File S2—Figure S6) demonstrate particles produced by SEC are concentrated in sEV fractions 8 and 10, and non-sEV fraction 13. UC + SEC resulted in earlier peak particle elution (i.e., sEV-enriched fraction 7) and additional peaks in non-sEV fractions 12, 13 and 15.

Following on from this, all methods were assessed for particle yield normalised to mL of plasma in case the order in which these methods are performed affects the efficiency of particle extraction from plasma. Pooled fraction analysis of the four methods showed a significant increase in particle concentration in samples resulting from the UC + SEC method compared to the SEC + UC/UF methods; however, there was also a general trend of increased particle number compared to the UC + SEC + UF method (Supplementary File S2—Figure S7A). The overall particle yield in each fraction 7–10 pool was significantly higher with the UC + SEC method compared to all other methods (** *p <* SEC + UC and UC + SEC + UF methods; *** *p <* SEC + UF method) (Supplementary File S2—Figure S7B). However, the particle yields per mL of plasma were not significantly different between all methods, although the variability was greatest by the SEC + UF method, while also displaying a general trend of increased particle yield by this method (Supplementary File S2—Figure S7C).

To estimate the purity of samples prepared using each method, the number of particles per µg of protein in sEV-enriched fraction 7–10 were calculated, as determined by micro BCA assay (Supplementary File S2—Figure S8A,B). The UC + SEC method contained, on average, more particles/µg protein than all other methods, and was significantly greater than the SEC + UF method in this respect (*p <* 0.05). The purity estimate of the SEC + UF method, however, was the least variable of all the methods.

#### 3.2.2. Size Distribution Profiles

**Figure 3:**
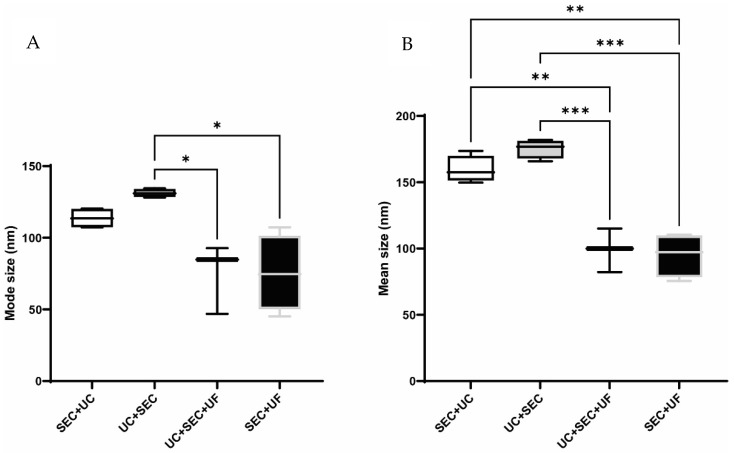
Mode (**A**) and mean (**B**) size distributions of pooled sEV fractions by all methods. In both mean and mode size analysis, UC + SEC particles were significantly larger than those obtained by the UC + SEC + UF and SEC + UF methods (UC + SEC + UF, *n* = 3; all other methods, *n* = 4; error bars ± SEM; * *p* < 0.05, ** *p* < 0.01, *** *p* < 0.001). The mean size of particles obtained by the SEC + UC method were significantly larger than the UC + SEC + UF and SEC + UF methods.

The order in which enrichment methods are performed may affect the size distribution of the sEV populations obtained, potentially introducing bias towards an sEV subtype of a specific size range. To observe the effect that the order and type of enrichment method has on particle size distribution, the mean and mode size ranges for particles produced by all methods were determined and are shown in Figure 3. These methods produced sEV-enriched samples with particles that fell within the sEV (diameter < 200 nm) range. However, the UC + SEC method produced particles that were significantly larger in mode and mean size than those produced by the UC + SEC + UF and SEC + UF methods (mode size * p < 0.05; mean size *** *p* < 0.001). The SEC + UC method also contained particles significantly larger by mean size than the UC + SEC + UF and SEC + UF methods (** p < 0.01).

To assess whether performing UC before or after SEC affected the size of the EV populations obtained, the size of particles in individual fractions resulting from UC + SEC and SEC were compared to pooled samples resulting from the UC + SEC and SEC + UC methods. The mean sizes of particles in the individual sEV-enriched fractions produced by SEC were of diameter ~150 nm, whereas those produced by UC + SEC were of diameter ~175–190 nm (Supplementary File S2—Figure S6B). Similarly, the mode sizes of particles produced in the SEC sEV-enriched fractions ranged from diameter ~96–110 nm, whereas the sEV-enriched fractions produced by the UC + SEC method contained particles with mode sizes of diameter ~116–167 nm (Supplementary File S2—Figure S6C). NTA of the pooled sEV-enriched fractions (Figure 3A) was consistent with this size difference, with the samples resulting from the SEC + UC method enriching for particles of mode size ~107–120 nm, and the UC + SEC fractions 7–10 pool showing enrichment for particles of mode size ~130–135 nm. Similarly, mean particle sizes resulting from SEC + UC and UC + SEC 7–10 pooled sEV were 150–174 nm and 166–182 nm, respectively (Figure 3B).

### 3.3. Transmission Electron Microscopy (TEM)

Small EV particles were visualised in pooled sEV fractions from each method to assess whether (a) all methods enriched for similar populations of EVs, and (b) whether concentration of samples by UF was comparable to the UC + SEC and SEC + UC methods. Small EVs ranged from ~50–150 nm and were distinguishable by their distinct cup- or round-shaped morphologies (Supplementary File S2—Figure S9A,B) [44]. Samples resulting from the SEC + UC method contained smaller particles than samples resulting from all other methods. Wide-view images visualised numerous large (>150 nm) particles in the samples produced by the UC + SEC + UF and SEC + UF enrichment methods, whereas the UC + SEC and SEC + UC methods showed particles in a smaller size range (<150 nm) (Supplementary File S2—Figure S9A). Small EV particles were not visualised in non-sEV-enriched fractions 11–16 (Representative non-sEV TEM images are available in Supplementary File S2—Figures S10 and S11). A microvesicle of ~700 nm diameter was observed in non-sEV pooled fractions 11–16 by the UC + SEC method, and soluble proteins and a few amorphous particles visible in non-sEV pooled fractions 11–16 by the SEC + UC method (Supplementary File S2—Figures S10 and S11).

### 3.4. Total Protein Quantification

Micro BCA assay was used to determine the total protein yield in samples enriched for sEV prior to MS sample preparation. SEC + UF produced samples with the highest concentration of protein (~40–150 µg/mL), but was the most variable (Supplementary File S2—Figure S9B). All other methods produced protein yields of <40 µg/mL. Total protein determination was used to calculate the volumes of samples required for protein digestion prior to MS analysis.

### 3.5. Mass Spectrometry (MS)-Based Protein Identification Results

#### 3.5.1. UC + SEC Enrichment Method Resulted in the Highest Number of Protein IDs

The protein content of EVs and sEVs are of paramount importance in biomarker studies. Therefore, the effect that the various enrichment methods in this study have on overall sEV protein ID, the level of contamination with non-sEV proteins from each enrichment method, and the consistency of results with online databases of known sEV proteins, were determined by MS analysis in DDA mode. The total number of protein IDs at 1% FDR is shown in Table 1. Protein IDs were also processed to include 5% FDR analysis at the pep tide level, and a minimum of two peptides per protein. All raw files (wiff/.wiff.scan) and ProteinPilot output files (group) have been deposited to the ProteomeXchange Consortium via the PRIDE [1] partner repository (http://www.proteomexchange.org/, accessed on 15 March 2022) with the dataset identifier PXD032313.

**Figure 4:**
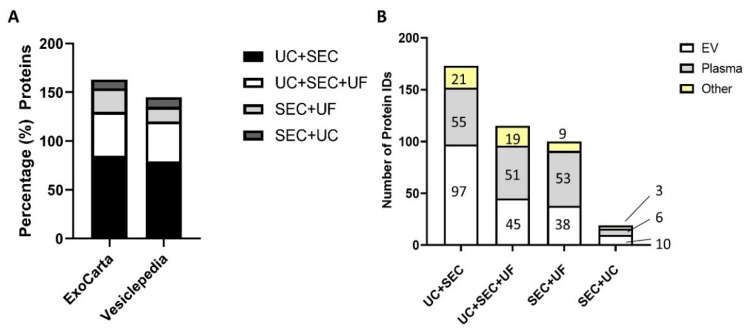
(**A**) Percentage of top 100 EV proteins (Vesiclepedia and ExoCarta) identified in sEV enriched samples by all methods. (**B**) Summary plot of mapped EV and non-EV proteins in the four methods under study identified in the Vesiclepedia complete database and plasma control. EV proteins = identified in samples and Vesiclepedia database; Plasma = identified in plasma and sEV-enriched sample; Other = identified in sEV-enriched sample only.

Small EV enrichment using the UC + SEC method resulted in the highest number of identified proteins. The UC + SEC method enabled identification of the highest number of the ‘top 100′ EV proteins from the online databases ExoCarta and Vesiclepedia (28 and 31, respectively) (Figure 4A). While SEC + UF had the third highest number of total protein IDs, only eight and six EV proteins were identified from ExoCarta and Vesiclepedia, respectively. Figure 4B shows the percent of the total number of proteins identified in the top 100 EV protein list with all of the sEV enrichment methods under study. The complete list of proteins can be found in Supplementary File S2 (Tables S1 and S2). Only one sEV protein, Galectin-3 binding protein (LGALS3BP), was identified in all enrichment methods and not in the plasma control group. The tetraspanin CD9 was identified in samples resulting from the UC + SEC, UC + SEC + UF, and SEC + UF methods, and not detected from those prepared using the SEC + UC method. CD81 was present in samples from the UC + SEC and UC + SEC + UF methods, and not detected in those prepared by the SEC + UC and SEC + UF methods. Notably absent from samples prepared using all of the sEV enrichment methods were the sEV markers tumour susceptibility gene 101 (TSG101) and flotillin-1 (FLOT-1). The UC + SEC method also resulted in the best sequence coverage (%, 95 confidence) for each protein ID in the top 100 EV list, where the protein was identified by more than one method (see Supplementary File S1).

Proteomic comparison of the samples prepared using the UC + SEC method with the complete Vesiclepedia protein database led to the identification of 138 proteins that were annotated in Vesiclepedia, while 21 were only identified in samples prepared using this sEV enrichment method (Supplementary File S2—Figure S12A). Gene ontology analysis of these 21 unique proteins using PantherGO resulted in the identification of gene families associated with vesicle-mediated transport (GO:0006897; GO:0009987; GO:0006900), plasma membrane (GO:0005886), and cell–cell recognition (GO:0008037; GO:0009987) (see Supplementary Files S3 and S4 for PANTHERGO gene ontology terms). Samples prepared using the UC + SEC + UF method shared 12 of the 21 unique proteins identified in samples prepared using the UC + SEC method, including those associated with vesicle-mediated transport and cell communication (GO:0006897; GO:0009987; GO:0006900).

To determine the total number of known EV proteins resulting from each enrichment method, only mapped (fully annotated) proteins were compared to the Vesiclepedia database and plasma control. Small extracellular vesicle enrichment using the UC + SEC method resulted in the highest number of EV proteins annotated in the Vesiclepedia database, but also identified the highest number of plasma proteins (Figure 4B). Samples prepared using all of the methods under study resulted in the identification of 51–55 plasma proteins, except for the SEC + UC method, which led to the identification of only six plasma proteins.

Small extracellular vesicles have been implicated in numerous signalling pathways related to the pathogenesis of disease and cancer metastasis. Therefore, the sEV isolation and enrichment methods were also assessed for the enrichment of proteins involved in pathways known to be associated with sEVs (Supplementary File S2—Table S4). Proteins involved in the cadherin, p53, Wnt, the Alzheimer disease-presenilin pathway, and the cholecystokinin receptors (CCKRs) map signalling pathways were increased compared to the plasma control in all methods except for the SEC + UC method. Proteins involved in the integrin signalling pathway were increased in all methods compared to plasma, while proteins involved in the Huntington’s disease pathway were increased only when the UC + SEC and SEC + UF methods were used. The use of the UC + SEC + UF method resulted in the highest enrichment of proteins involved in the cadherin, Wnt, CCKRs, Alzheimer’s, and p53 pathways.

#### 3.5.2. Depletion of Abundant Plasma Proteins following sEV Enrichment

**Figure 5:**
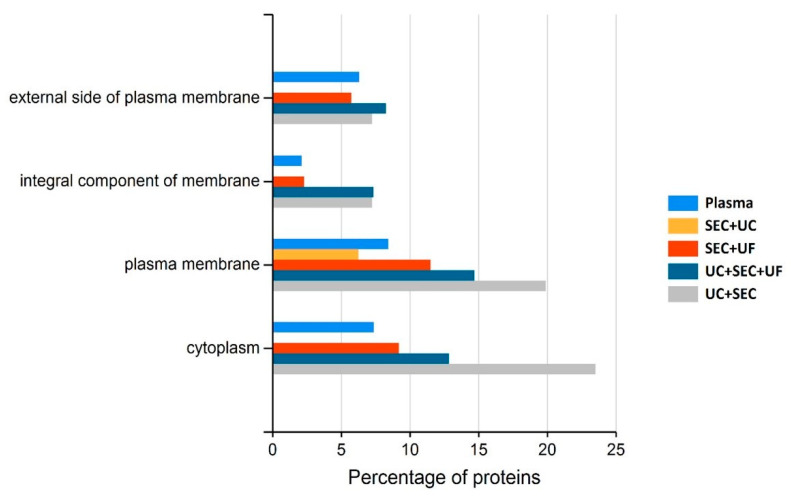
Functional enrichment analysis (cellular component) of detected proteins in plasma processed using one of the four methods for sEV protein enrichment and plasma control.

The optimal MS data acquisition for sEV-enriched samples relies on the successful depletion of highly abundant plasma proteins, which may impede the detection of less-abundant sEV proteins. To assess the presence of plasma proteins resulting from each method of enrichment and whether concentrating samples by adding/substituting UF improved depletion of highly abundant plasma proteins, each method was subjected to functional enrichment analysis. As sEV proteins are often associated with the plasma membrane (PM) and the internal environment of the cell, functional enrichment analysis for proteins associated with the PM and cytoplasm was performed using the online software tool, FunRich. Proteins detected in each method of enrichment and a plasma control were compared with respect to cellular component (see Figure 5). The SEC + UC method enriched for proteins associated with the PM but were depleted/not detected in all other categories. The UC + SEC method resulted in a 2.5-fold increase in proteins associated with the PM, proteins considered as part of the integral component of the membrane, and the cytoplasm compared to the plasma control. The UC + SEC, UC + SEC + UF, and SEC + UF methods did not significantly alter the number of proteins associated with the external side of the plasma membrane, although the UC + SEC + UF method outperformed the other methods in this case.

To further explore the presence of plasma proteins in the sEV samples derived from each method of enrichment, gene ontology pathway analysis was carried out using PANTHERGO. Pathway analysis revealed that 44% of the proteins identified in samples prepared with the SEC + UC method were associated with blood coagulation, compared with 24% when the UC + SEC was used, 29% when the SEC + UF method was used, 21% when the UC + SEC + UF method was used, and 39% in the plasma control (Supplementary File S2—Table S4). A Venn diagram and table of proteins common to all methods of enrichment can be found in Supplementary File S2 (Figure S13 and Table S5).

Finally, using peptide ID analysis, depletion of abundant plasma proteins was compared for each method. sEV-enriched samples were assessed by comparing the number of peptides identified for known abundant plasma proteins to a plasma control sample [45]. The proportion of peptides in sEV enrichment methods versus plasma control peptides was calculated for each plasma protein (see Table 2). All methods resulted in increased number of peptides identified for IgA/IgM, while only those methods utilising UC first showed a 3 to 3.67-fold increase in peptide ID for haptoglobin as compared to the plasma control. The use of the SEC + UF method increased the number of peptides identified for the low-density lipoprotein (LDL), whereas all other methods showed fewer LDL peptide IDs. The depletion of albumin peptides was highest in the UC + SEC and UC + SEC + UF methods.

## 4. Discussions

We have demonstrated that sEV enrichment from plasma for the purpose of downstream proteomics can be achieved by UC, SEC and UF performed in selected combinations and different orders. The UC + SEC method resulted in the largest number of identified EV markers, while also reducing the number of peptide IDs of 9 out of 14 abundant plasma proteins. The addition of UF did not improve the purity of the samples based on peptide ID of common blood contaminants; however, it did result in a significant loss of common EV markers and sEV-related proteins, as described in the enrichment analysis (Section 3.5.1).

### 4.1. SEC May Lead to Variable Fractionation of sEV and Plasma Proteins

The process of fractionation using commercially available SEC columns, qEV original (Izon Science, Christchurch, New Zealand), involves the manual collection of 0.5 mL fractions. Therefore, the reproducibility of this process is likely to be user-dependent, especially when performing two or more fractionations simultaneously to improve sample throughput. This was demonstrated in a recent study where higher inter-sample variation occurred using SEC columns as opposed to commercially available sEV precipitation kits, exoEasy (QIAGEN) and ExoQuick (System Biosciences) [42]. Additionally, if high sample purity is of specific importance to the study, then WB for selected plasma proteins should be performed prior to pooling the various SEC fractions. In this study, the amount of BSA present in the UC + SEC pooled sample did not appear to have a detrimental effect on the number of sEV proteins identified compared to the other methods. The consequence of omitting an sEV fraction due to the increased albumin presence must be weighed against the loss of a significant portion of the sEV particles, which may have a deleterious effect on the number of protein IDs or quantitation of proteins if performing quantitative proteomics studies [30].

### 4.2. The Addition of UF Alters Size Distribution by Reducing the Mean and Mode Size of Particles

The order of sEV enrichment steps within a method affects the size of the sEV particles obtained. Although sEVs are still within the expected size range, it is of interest to know whether performing UC prior to SEC further enriches the sample in a specific subpopulation of sEV—e.g., in the upper size range—thus introducing bias. Small EV size differences have been used successfully as a prognostic tool for disease and thus are of interest in biomarker studies [8,46]. It has already been established that UC performed independently on plasma co-isolates particles in the 20–250 nm size range, while SEC isolates particles of diameter 20–200 nm [47]. This is supported by a separate study showing that EVs isolated from cell culture media with a UF+SEC method contained fewer particles of size > 200 nm than if only UC was performed [32]. In this study, the addition of UF significantly reduced the size of particles obtained compared with other methods. Unfortunately, UF also resulted in a significant reduction in particle concentration and yield, which poses a question of feasibility for the number of downstream analyses than can be performed while incorporating this method.

UC can lead to both sEV and protein aggregation as a result of extended periods of high centrifugal force, whereas commencing sEV enrichment with SEC bypasses this issue [11,48,49,50,51]. Incorporating UC as a method of sEV enrichment without UF is therefore likely to co-isolate larger particles initially, and may explain their increased presence in the sEV-enriched samples resulting from the UC + SEC and SEC + UC methods in this study. The increase in size may be due to several reasons: the isolation of larger EV populations by commencing the enrichment process with UC as has been previously reported [47], aggregation of sEVs, or plasma protein aggregates. Indeed, the plasma proteins detected in samples enriched by the UC + SEC and UC + SEC + UF methods contained more immunoglobulins and haptoglobin than the other two methods, and hemoglobin subunit beta was detected in greater quantities by the UC + SEC method than all other methods. The TEM images did not show a noticeable size difference in sEV particles between the methods studied here, but instead showed an increase of aggregates and non-sEV material in sEV-enriched UC + SEC fractions 7–10 and the UC + SEC + UF fractions 7–10. Increased presence of these aggregates would result in different particle elution profiles, as was observed when comparing SEC to UC + SEC individual fractions by NTA. The presence of non-sEV particles in the sEV-enriched samples resulting from UC + SEC needs to be taken into account when performing particle size analysis. The particle size analysis results should be interpreted with caution and always compared to particle visualisation by TEM or similar techniques.

The formation of sEV and protein aggregates during UC should be considered carefully if the intent is to follow UC with further enrichment by SEC. Disruption of aggregates prior to SEC may yield purer fractions containing lower amounts of albumin or other unwanted plasma proteins. The reduction of aggregate formation may also result in better separation of EV particles and improve sEV particle yield, given that optimal fractionation depends almost entirely on particle size. The addition of UF reduced mean particle size to ~100 nm average diameter, suggesting that this method is effective for removing larger particles/aggregates resulting from UC.

BSA is ~3 nm in diameter; therefore, the increased BSA contamination of the sEV-enriched fraction 7 in the UC + SEC method indicates either significant BSA protein self-aggregation or sEV aggregation with surface-bound BSA, causing it to elute in a fraction that theoretically should have contained particles of ~ 100–200 nm in diameter. Methods to disrupt aggregates should be employed with care in regard to downstream analyses. Digestion of unwanted proteins by treating with proteases may compromise proteins bound to the surface of sEVs, or to the external domains of membrane proteins [26,52]. Likewise, extended periods of sonication could degrade sEV proteins or cause leakage and loss of internal sEV components into the external milieu [53].

### 4.3. Small EV-Associated Pathways Display Variable Enrichment Dependent upon sEV Isolation Method

As the molecular cargo of sEVs is essential to cell–cell communication in many molecular signalling pathways involved in health and disease, the enrichment of proteins associated with signalling pathways from plasma is critical to ongoing research in these areas. Small extracellular vesicles have been implicated in the regulation of neurodegenerative diseases, such as Huntington’s and Alzheimer’s diseases, and have been shown to promote metastasis via the Wnt, cadherin and integrin signalling pathways in various types of cancer [54,55,56,57,58]. Similarly, sEV release from cells in response to DNA damage is under the control of p53-driven gene expression, and sEV-derived miRNA are essential for mutant p53-associated oncogenesis [59]. Furthermore, the stimulation of CCKRs have been shown to increase the release of EVs by more than two-fold in human and mouse trophoblast cell lines [60]. The increased presence of proteins associated with these pathways in sEV-enriched samples suggests that it is possible to isolate these sEV-populations in peripheral plasma, which could be of utility in clinical studies. However, the enrichment of proteins involved in these pathways was not consistent across each method in this study. Overall, the UC + SEC method was the most consistent in identifying proteins involved in all sEV-related pathways assessed in this study. However, if the aim is to isolate vesicles for a specific pathway of interest, thought should be given to the choice of isolation method, as this will likely affect the success of enrichment for the desired subset of proteins.

### 4.4. The Method of sEV Enrichment Has a Direct Impact on Protein ID

In protein biomarker studies, the quality of the sample is paramount to the success of the project. We have shown that the sEV enrichment strategy is critical for the depletion of plasma proteins and the optimal identification of sEV-associated proteins. The UC + SEC method was found to provide the best particle yield, purity, the largest number of protein identifications, and sEV marker IDs. This is consistent with a previous study that determined that the UC + SEC method was an efficient method for producing the optimum particle yield [23]. The addition of UC or UF after SEC drastically reduces the particle concentration of sEV-enriched samples compared to similar studies that performed SEC alone using the qEV 70 nm SEC columns utilised in this study, and reduces the protein ID of sEV markers [29,61]. However, the purity estimate in this study indicates that the UC + SEC method results in improved particles/µg protein than a study that evaluated five different methods of sEV enrichment, including qEV 70 nm SEC columns and optiprep density gradient UC [42]. The SEC + UC method was found to be the most effective technique for reducing the number of plasma proteins based on peptide ID, although blood coagulation components still accounted for over 44% of the total number of proteins. This is likely due to the SEC + UC method yielding the lowest number of proteins out of the four sEV isolation and enrichment methods studied here. While 251 proteins were identified in samples resulting from the SEC + UF method (1% FDR), the number of top 100 sEV marker proteins represented only 3.2% of the total number of proteins identified in this method. In comparison, only 81 proteins were identified in total by the SEC + UC method, but the number of top 100 EV marker proteins represented 4.9% of the total. Finally, the top 100 EV markers identified when the UC + SEC method was used accounted for 8.9% of the total number of proteins. It is therefore clear that the discernment of EV or sEV proteins from the total number of proteins identified is the key to determining whether the enrichment method was effective in a) enriching for sEVs and, b) depleting the sample of the highly abundant plasma proteins contained in the original biofluid. Additionally, the UC + SEC method improved the number of proteins identified as sEV proteins by ExoCarta and Vesiclepedia, compared to other studies that used at least one of the commercially available kits, ExoSpin and ExoQuick, to isolate sEV from human plasma [42,62]. As a species-specific database for plasma sEVs has not yet been established, and many of the proteins identified by each method are yet to be fully characterised, there are potentially many more sEV proteins present than we were able to match to existing EV databases [63]. For example, 247 proteins were identified when the UC + SEC method was used, of which 173 were mapped (fully annotated). The 21 proteins unique to sEVs that were identified in samples prepared by the UC + SEC method that were not listed in the Vesiclepedia database are also an example of this, as GO analysis evidenced their association with sEV-related processes. These proteins, however, are currently unreviewed in Uniprot (https://www.uniprot.org/, accessed on 10 June 2021).

Interestingly, FLOT-1 and TSG101, which are frequently used as EX markers, were not detected by MS in any of the isolation and enrichment methods studied here. The absence of FLOT-1 in the sEV-enriched samples produced by all of the studied methods was confirmed by WB. The enrichment of FLOT-1-positive EX may therefore not be possible using the methods described in this study, or these vesicles have low abundance in plasma. However, it should be noted that a study that profiled the bovine milk EX proteome identified TSG101 and FLOT-1 by WB in samples processed using UC and SEC as standalone enrichment methods [64]. FLOT-1 has previously been identified in human plasma by WB analysis following immunocapture of CD9- and CD81-positive EXs [7]. The same study also demonstrated that TSG101 was not present in plasma EXs. Therefore, FLOT-1 may be cell-type specific and not released in large quantities into systemic circulation [7]. In a more recent methodological evaluation study using ExoQuick, exoEasy, SEC and OptiPrep, MS also failed to detect FLOT-1 in human plasma [42]. Dong and colleagues (2020) detected FLOT-1 in EXs isolated from human plasma by UC and SEC + UF methods after performing WB analysis [20]. However, the amount of protein loaded to achieve this was ten times that of the amount used in the current study. A separate study utilising quantitative proteomics to analyse various exosomal preparations noted that FLOT-1 was not enriched in 100,000× *g* pellets compared to 2000- and 10,000× *g* pellets, thus the authors stated it could not be considered as a marker of sEVs nor EXs [65]. Whether a targeted EX enrichment strategy would be better suited to study FLOT-1-positive EX populations in plasma is unclear, but the four isolation and enrichment methods utilised in this study were unsuccessful in isolating sEVs that contained FLOT-1 in sufficient amount for detection by WB or MS. An alternative approach would be to use direct immunocapture of specific sEV markers of interest to further explore EV subpopulations, as non-specific methods isolate a heterogenous mixture of EV and non-EV particles, as is the case in this and many other studies. The data presented here merely provides an overview of the feasibility of performing MS analysis of sEV using different methods of enrichment and purification.

The identification of LGALS3BP in all sEV-enrichment methods utilised in this study is a novel finding. LGALS3BP has previously been identified in human and animal studies from plasma or cell culture-conditioned media using UC with iodixanol gradient or sEV isolation kits (ExoSpin/ExoQuick) [62,66]. Zhang and colleagues (2019) were the first to link the enrichment of LGALS3BP, a sialoglycoprotein, to the EV subclass exomeres (diameter ~35 nm) by using asymmetric field flow fractionation of cancer cell lines [8]. Subsequently, exomeres were successfully isolated by a modified sequential UC method, thus the ease in which they can be studied has improved [67]. In addition to being linked to glycosylation modulation and protein folding, exomeres are also enriched with metabolic proteins and proteins associated with neurological diseases, such as Alzheimer’s and Parkinson’s diseases [8,67]. Proteins associated with these disease pathways were indeed enriched in sEV samples produced in this study, as previously mentioned (Section 3.5.1). As exomeres have been found to co-isolate with sEV in this and other studies using a standard differential centrifugation method for enrichment, it is of interest as to whether the origin of many ‘sEV’ proteins may in fact be exomere-derived. While studies involving exomeres have provided valuable information regarding their characteristics, very little is known regarding their biogenesis, or their function in systemic circulation [68]. It is outside the scope of this project to determine the number of exomeres within our sEV-enriched samples, but future studies should be able to determine the proportion of exomeres in heterogenous EV and/or sEV samples, and whether they have any association with health and disease.

## 5. Conclusions

In this study, the method resulting in the optimum yield of sEVs, the highest purity, the most protein IDs with minimal plasma protein identification, and the highest possible identification of sEV-associated proteins was the UC + SEC method, followed by a modified FASP protocol. The SEC + UC method did not significantly alter the characteristics of the particles obtained compared with UC + SEC, and was comparable to the SEC + UF and UC + SEC + UF methods with respect to particle concentration and particle yield. However, SEC + UC was the least successful method for the ID of both EV and total proteins. If sample pooling is necessary, consideration should be given to performing WB analysis prior to pooling to confirm the relative abundance of albumin in sEV fractions.

Interestingly, we identified LGALS3BP in all enrichment methods and not in the plasma control sample. This protein is known to be enriched in exomeres, suggesting the co-isolation of this EV subtype in all methods utilised in this study. LGALS3BP warrants further investigation as a possible target protein for the direct immuno-capture (DIC) of exomeres, which would simplify exomere enrichment from blood plasma if desired [67].

It is now widely accepted that sEV populations differ depending on the cell type or biofluid of origin [7,8,66,69]. To our knowledge, this is the first study to identify a significant difference in the mean and mode sizes of sEVs depending on whether UC was performed before or after SEC. Numerous studies have provided size data for individual enrichment strategies, but did not compare the effects on size distribution following multiple enrichment methods performed in different orders [26,29,70]. It is useful to know that UF as an sEV sample clean-up method is not recommended for downstream proteomics analysis due to the significant amount of sEV protein loss that occurs, even with a low molecular weight cut-off filter, as was used in this study.

Importantly, an sEV database designed specifically for livestock has not yet been established. A species search is possible using ExoCarta and Vesiclepedia, but in *Bos taurus*, many of these proteins have been identified in milk, not plasma. Vesiclepedia currently has no listings for sEV proteins identified in the plasma of dairy cows. We have provided evidence that a significant portion of the top 100 and above sEV proteins listed in the predominantly human sEV databases, ExoCarta and Vesiclepedia, are also present in the plasma of cow species *Bos taurus*. This is a critical finding as it suggests the roles of sEVs are conserved across mammalian species and highlights their biological significance.

## Figures and Tables

**Table 1:** Number of proteins identified in sEV enrichment methods. Proteins at 1% FDR, peptides at 5% FDR, with minimum two peptides per protein.

Method	UC + SEC		UC + SEC + UF		SEC + UF		SEC + UC	
**FDR method**	1% FDR	5% FDR, 2 pep	1% FDR	5% FDR, 2 pep	1% FDR	5% FDR, 2 pep	1% FDR	5% FDR, 2 pep
**Number of proteins identified**	349	247	237	155	251	128	81	49

**Table 2:** Proportion of peptides identified in sEV enriched samples compared to a plasma control. Green indicates a decrease and red an increase in peptides identified by one of the four sEV enrichment methods compared to a plasma control (Plasma = 1).

Plasma Proteins	UC + SEC	UC + SEC + UF	SEC + UF	SEC + UC
Albumin	0.16	0.16	0.31	0.24
α1-Antitrypsin	0.31	0.19	0.38	0.08
IgA/IgM	6.25	6.25	5.75	1.50
Transferrin	0.00	0.19	0.00	0.00
Haptoglobin	3.33	3.67	0.00	0.00
α2-Macroglobulin	1.22	0.84	1.49	0.18
Fibrinogen	1.86	1.41	3.24	0.35
Complement C3	0.48	0.37	0.51	0.09
α1-Acid Glycoprotein (Orosomucoid)	0.00	0.00	0.00	0.00
HDL (Apolipoproteins A-I)	0.55	0.45	0.73	0.27
HDL (Apolipoproteins A-II)	0.50	0.50	0.50	0.00
LDL (mainly Apolipoprotein B)	0.29	0.13	1.95	0.00
Hemoglobin subunit alpha	0.88	0.75	0.00	0.00
Hemoglobin subunit beta	1.07	0.86	0.64	0.14

## Data Availability

The data presented in this study are available within this article or in Supplementary Files S1–S9. All raw MS files (.wiff/.wiff.scan) and ProteinPilot output files (.group) have been deposited to the ProteomeXchange Consortium via the PRIDE [1] partner repository (http://www.proteomexchange.org/, accessed on 15 March 2022) with the dataset identifier PXD032313.

## Supplementary Materials

The following supporting information can be downloaded at: https://www.mdpi.com/article/10.3390/proteomes10020019/s1, Supplementary File S1: collated MS results, seq coverage, peptide ID, mass errors; Supplementary File S2: production of StageTips; Supplementary File S3: UC + SEC PANTHERGO; Supplementary File S4: UC + SEC + UF PANTHERGO; Supplementary Files S5–S8: Protein pilot outputs for SEC + UC, SEC + UF, UC + SEC, UC + SEC + UF; Supplementary File S9: NTA and micro BCA CVs.

## Abbreviations

BCA	Bicinchoninic acid
BSA	Bovine serum albumin
CCKR	Cholecystokinin receptors
DDA	Data-dependent acquisition
DPBS	Dulbecco’s phosphate buffered saline
EV	Extracellular vesicle
EX	Exosome
FASP	Filter aided sample preparation
FDR	False discovery rate
FLOT-1	Flotillin-1
ID	Identification
LDL	Low-density lipoprotein
LGALS3BP	Galectin-3 binding protein
MS	Mass spectrometry
Non-EV	Non-extracellular vesicle
NTA	Nanoparticle tracking analysis
PBS	Phosphate buffered saline
PBST	Phosphate buffered saline and 0.1% Tween-20
PM	Plasma membrane
PPAI	Post-partum anoestrous interval
SDC	Sodium deoxycholate
SEC	Size-exclusion chromatography
sEV	Small extracellular vesicle
TEM	Transmission electron microscopy
TFA	Trifluoroacetic acid
TOF	Time-of-flight
TSG101	Tumour susceptibility gene 101
UC	Ultracentrifugation
UF	Ultrafiltration
WB	Western blot
