# Supplementary material for: A Comparison of Blood Plasma Small Extracellular Vesicle Enrichment Strategies for Proteomic Analysis
Source: Proteomes. 2022 Jun 1;10(2):19. doi: 10.3390/proteomes10020019 (PMC9229025; doi:10.3390/proteomes10020019)
Supplement: Supplementary file 1 [file proteomes-10-00019-s001.zip › Supplementary file S2.pdf]

Supplementary Figures

Western blot full images

S1. BSA (SEC)

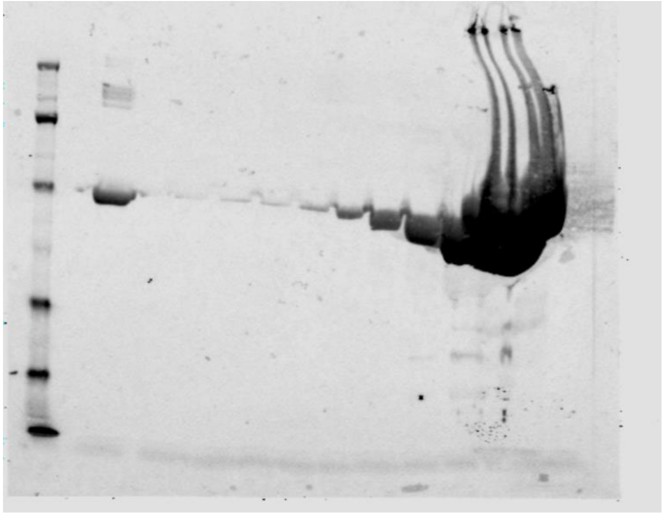

S2. BSA (UC+SEC)

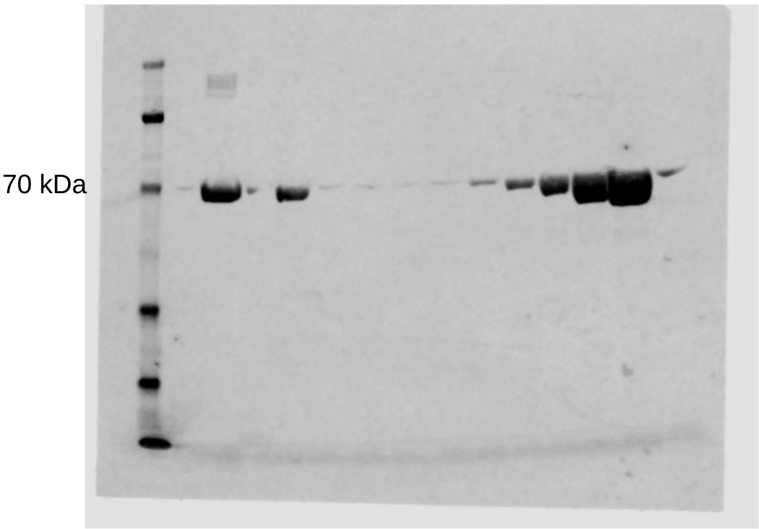

S3. FLOT-1

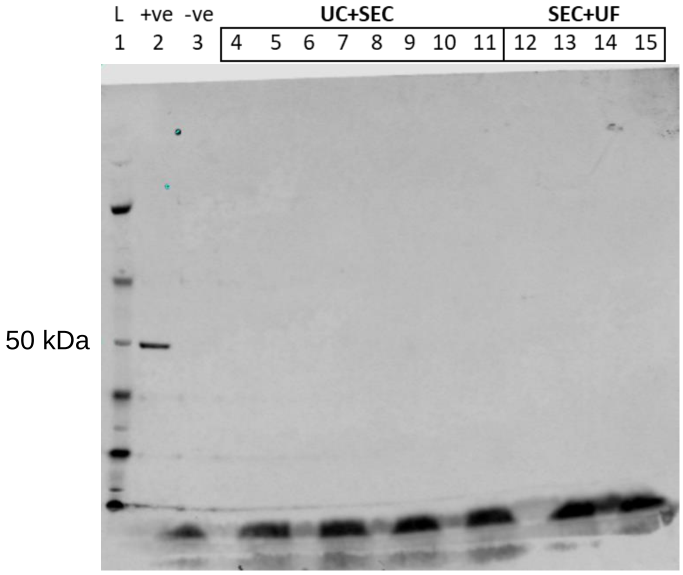

S4. FLOT-1

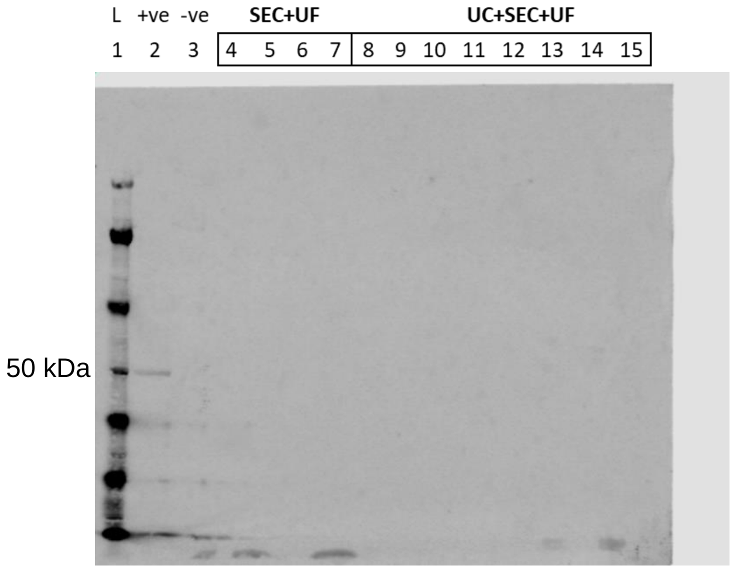

S5. FLOT-1

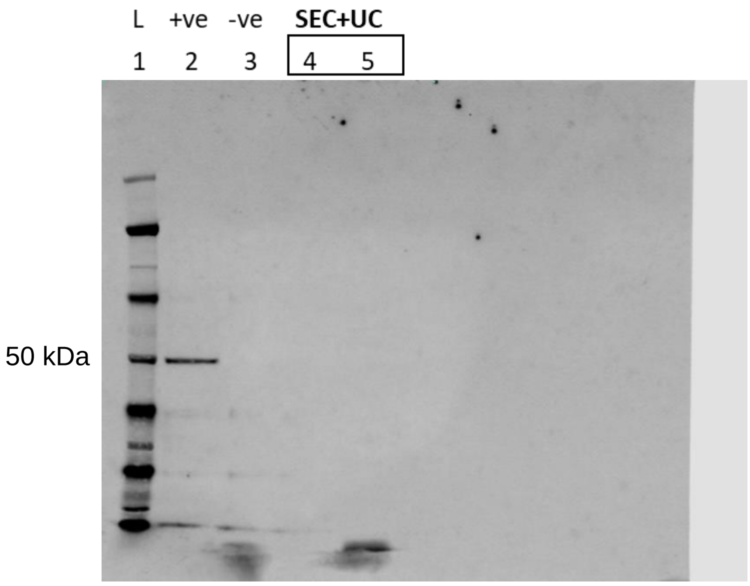

Figures S3 – S5: Western blot of Flotillin-1 (FLOT-1). L = ladder; +ve = positive control, 2.5 µg JEG-3 cell lysate; -ve = negative control, 5 µL de-ionized H_2O. A and B: As annotated for each method, even-numbered lanes = pooled sEV fractions 7 – 10 resulting from SEC. Odd-numbered lanes = pooled non-sEV fractions 11 – 16 resulting from SEC. C: Lane 4; pooled sEV 7 – 10 fractions for all replicates; lane 5 = pooled non-sEV fractions 11 – 16 for all replicates. Predicted molecular weight of FLOT-1 = 47 kDa.

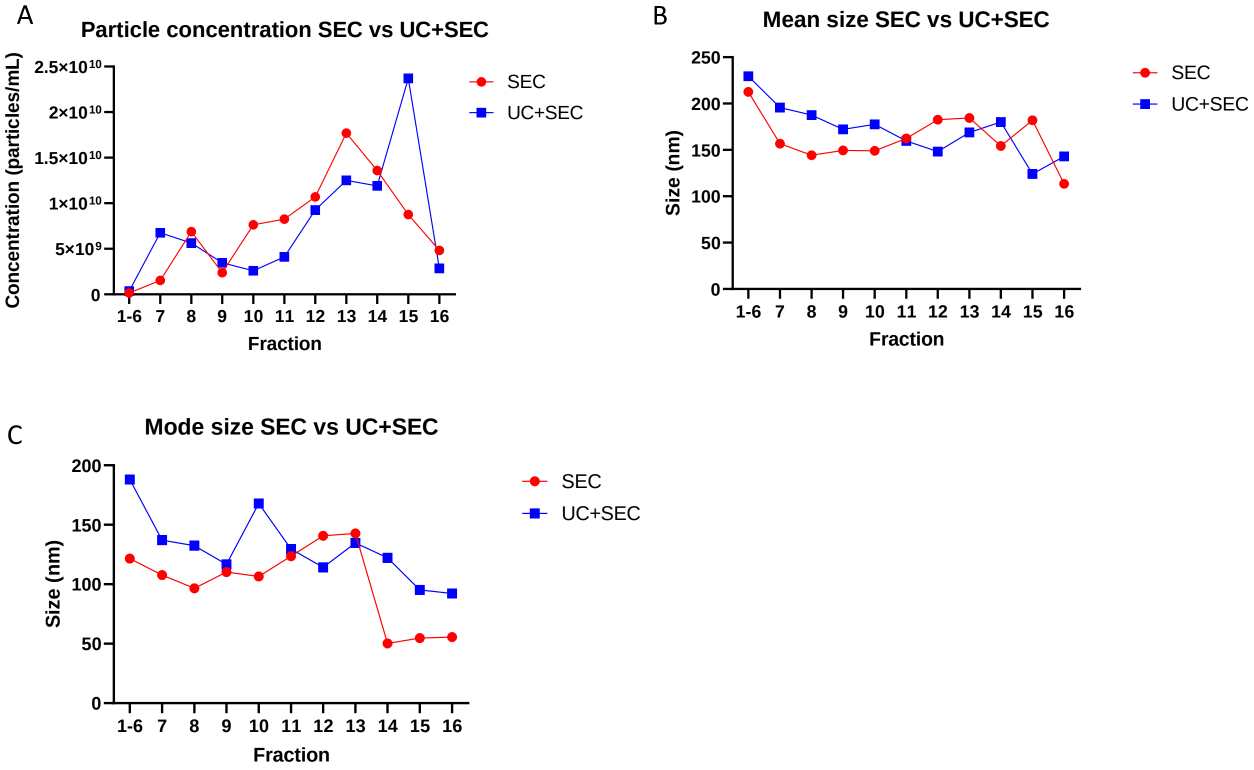

Figure S6: A) Particle concentration (particles/mL) distribution of SEC and UC+SEC fractions 1-6 (pool) and 7 – 16 individual fractions. Peak concentration of particles in sEV fractions 7 – 10 occurs in fraction 10 in SEC, and fraction 7 in UC+SEC. B) Mean and C) Mode size distributions of SEC and UC+SEC fractions 1 – 6 (pool) and individual fractions 7 – 16. All particles in sEV fractions 7 – 10 fell within small EV range (<200 nm). The mode size of SEC particles in sEV fractions 7 – 10 ranged from ~90 – 110. The mode size of UC+SEC particles in sEV fractions 7 – 10 ranged from ~116 – 167 nm.

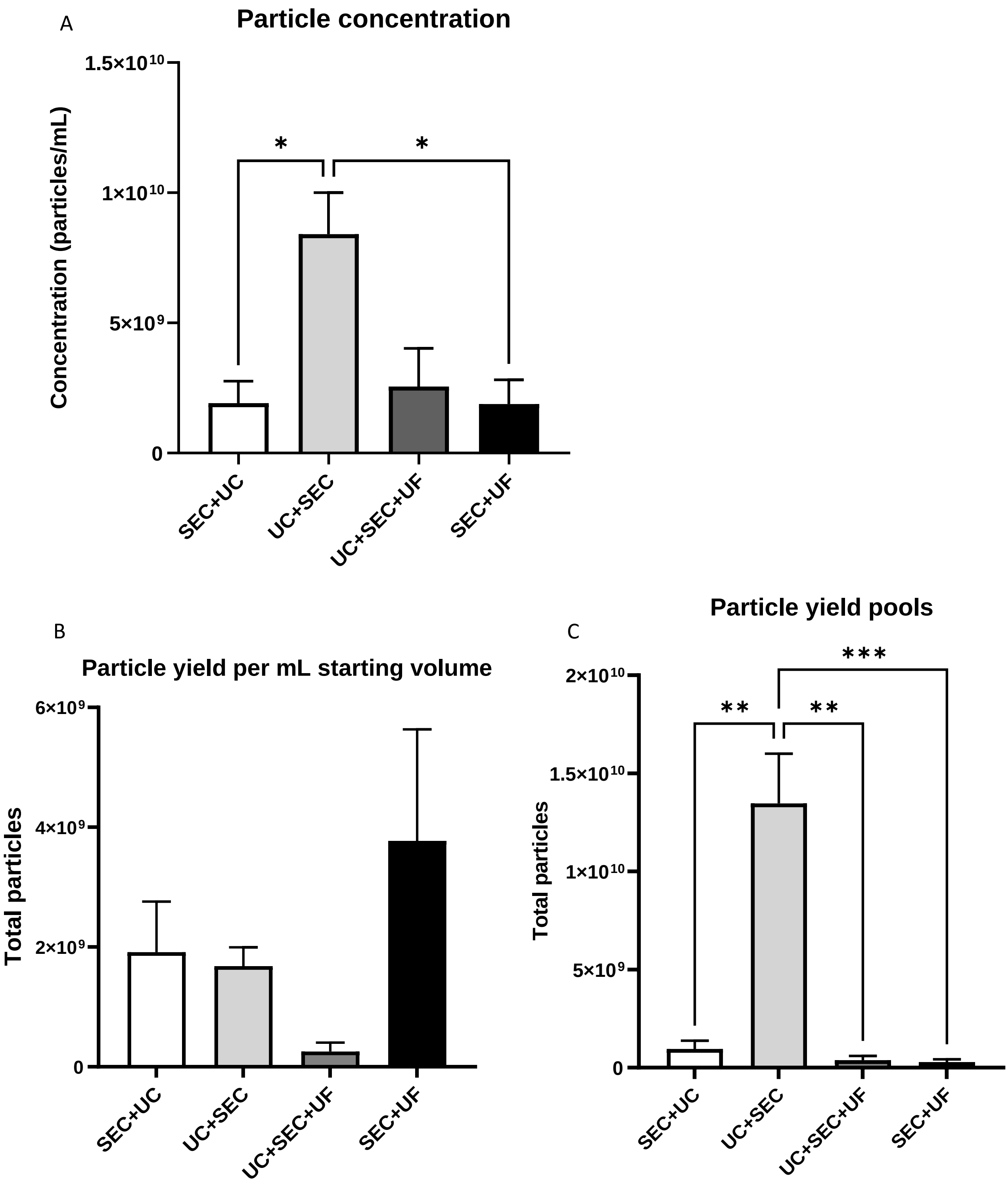

Figure S7: A) Particle concentration (particles/mL) in fraction 7 – 10 pools by all methods. UC+SEC > SEC+UC and SEC+UF. B) Particle yield (total particles) normalised per mL of starting volume of plasma, and C) particle yield (total particles) in fraction 7 – 10 pools by all methods. $^*p < 0.05$, $^{**}p < 0.01$, $^{***}p < 0.001$ (A-C: UC+SEC+UF, $n = 3$; all other methods, $n = 4$; error bars ± SEM).

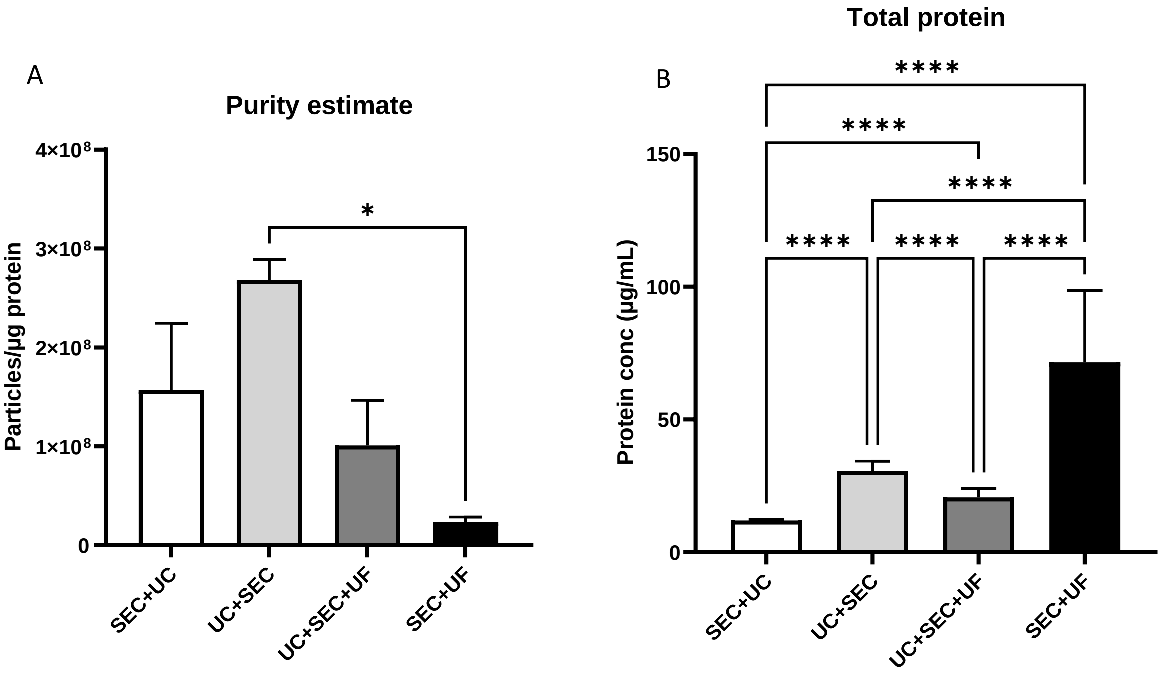

Figure S8: A) Purity estimate of particles obtained by all enrichment methods in pooled sEV fractions 7 – 10 (n = 3-4/method, error bars +/- SEM). B) Micro BCA assay results for sEV-enriched samples resulting from each method. Samples represent pooled sEV fractions 7 – 10. *$p < 0.05$, **$p < 0.01$, ***$p < 0.001$, ****$p < 0.0001$ (A: UC+SEC+UF, n = 3; all other methods, n = 4; error bars ± SEM; B: n = 4 all methods; error bars ± SEM).

TEM images

S9.

A

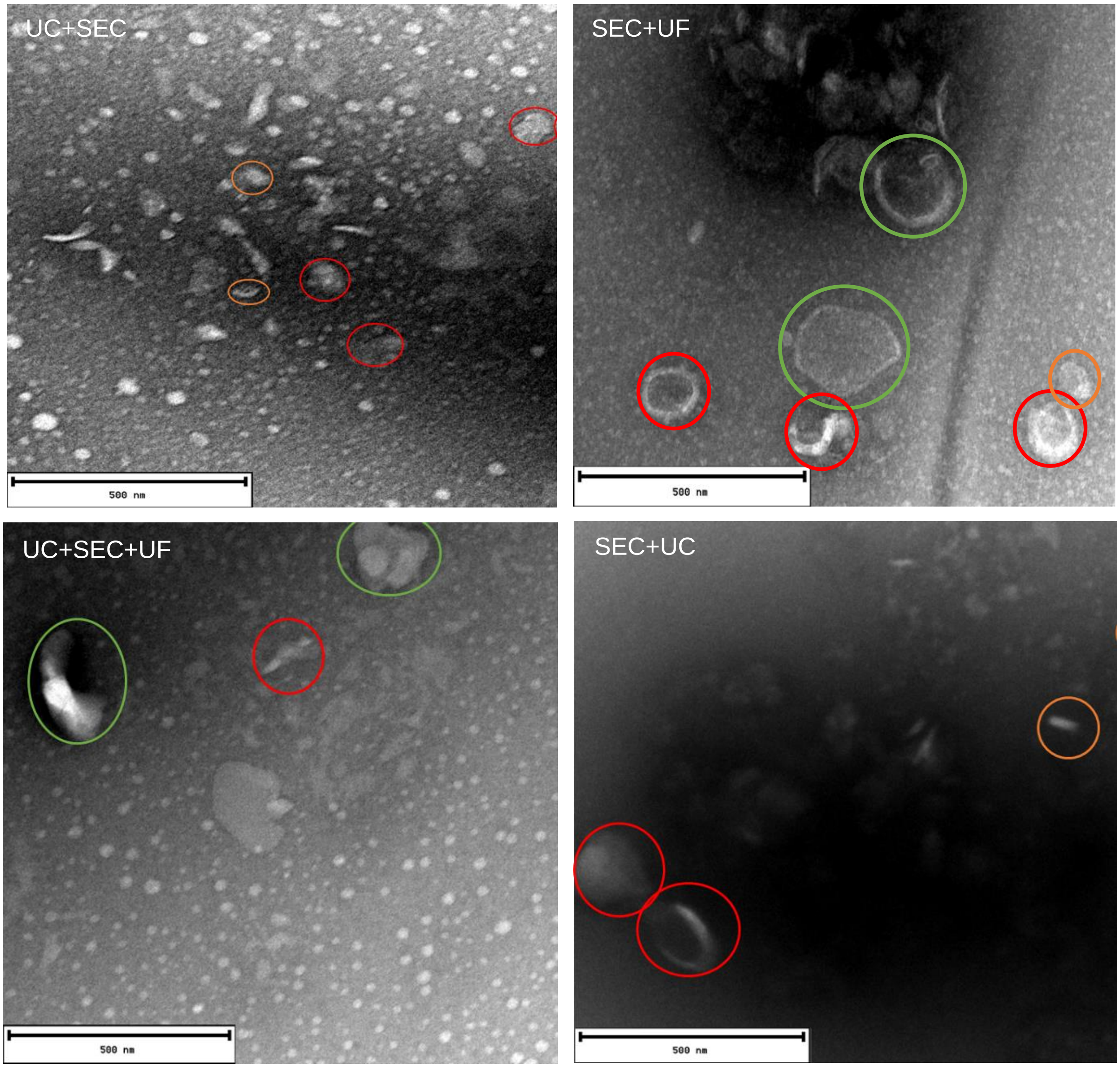

B

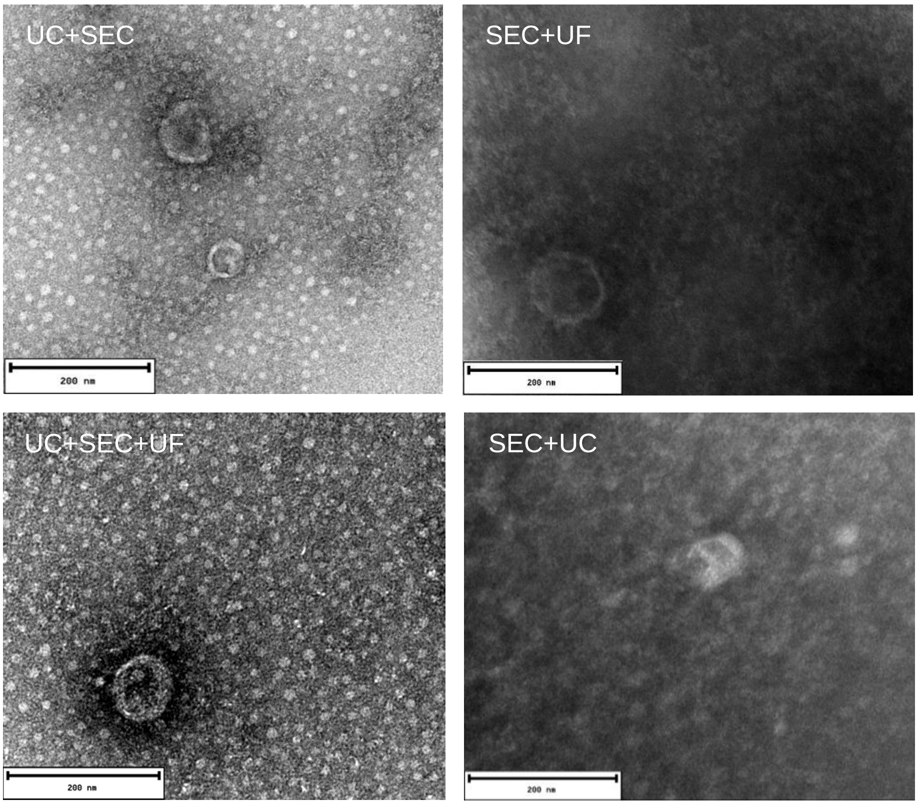

Figure S9A and B: Representative TEM images of pooled sEV fractions 7 – 10 by each of the four methods of sEV isolation and enrichment. **A:** Widefield view of sEV fractions 7 – 10. **B:** Single particle views of sEV fractions 7 – 10. Colour legend for circled particles in A: orange, < 75 nm; red, 75 – 150 nm; green, >150 nm.

S10. Non-sEV fraction pool 11 – 16 (UC+SEC). Scale bar = 500 nm.

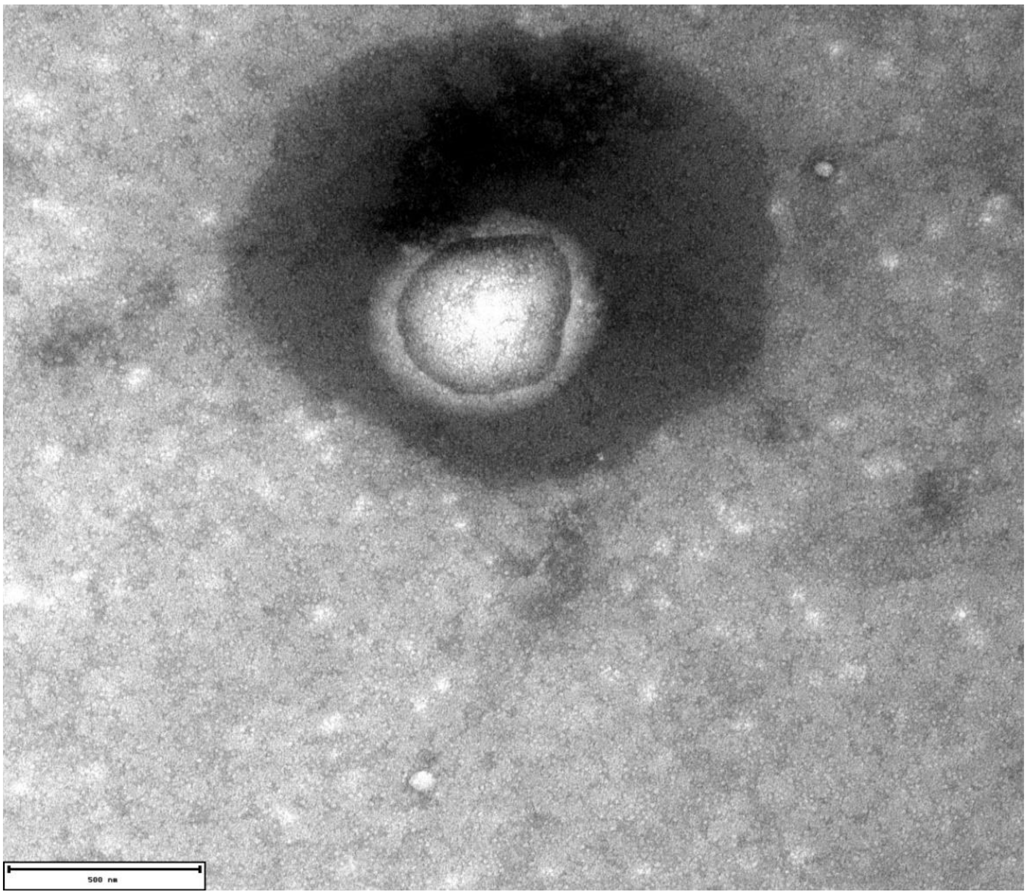

S11. Non-sEV fraction pool 11 – 16 (SEC+UC). Scale bar = 500 nm.

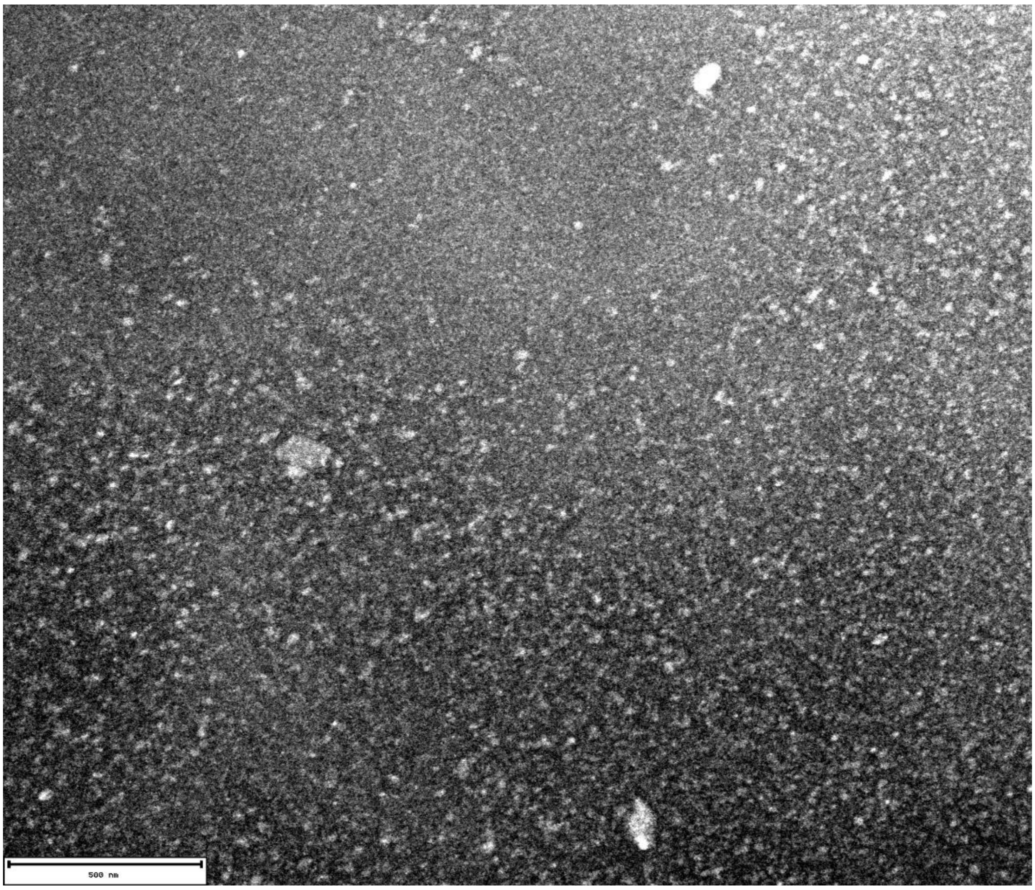

A

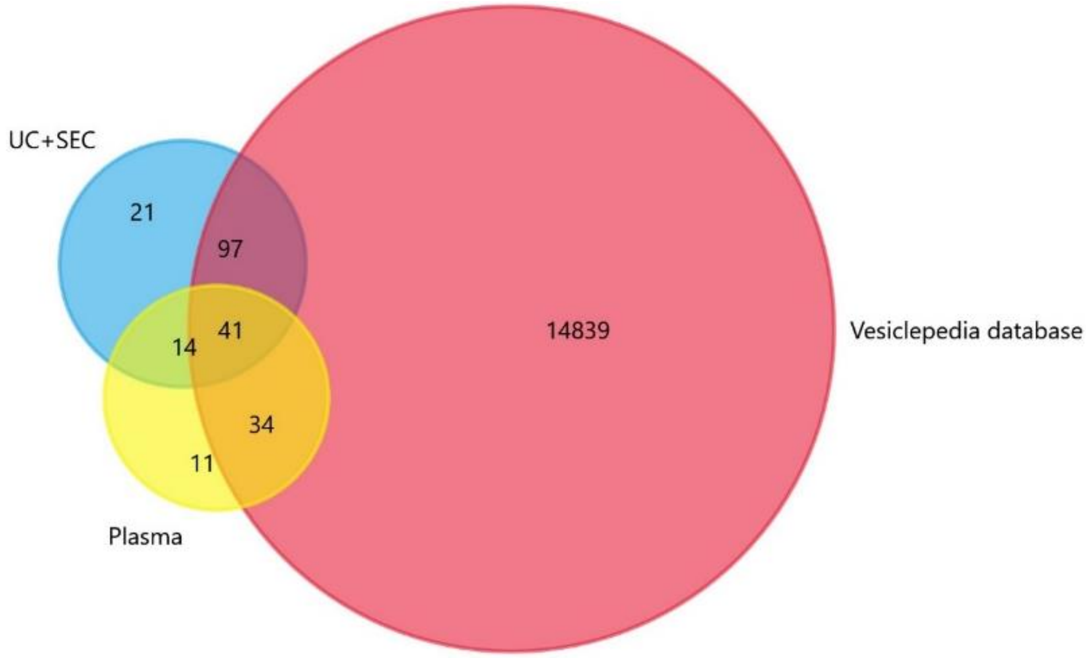
UC+SEC
21
97
41
14
34
11
14839
Vesiclepedia database
Plasma

B

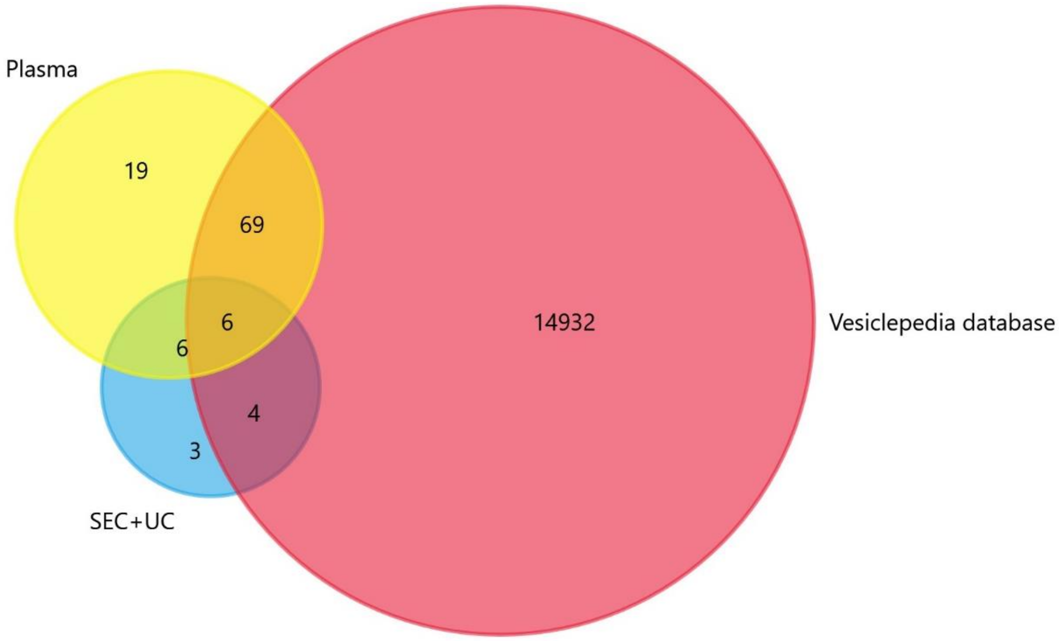
Plasma
19
69
6
6
4
3
14932
Vesiclepedia database
SEC+UC

C

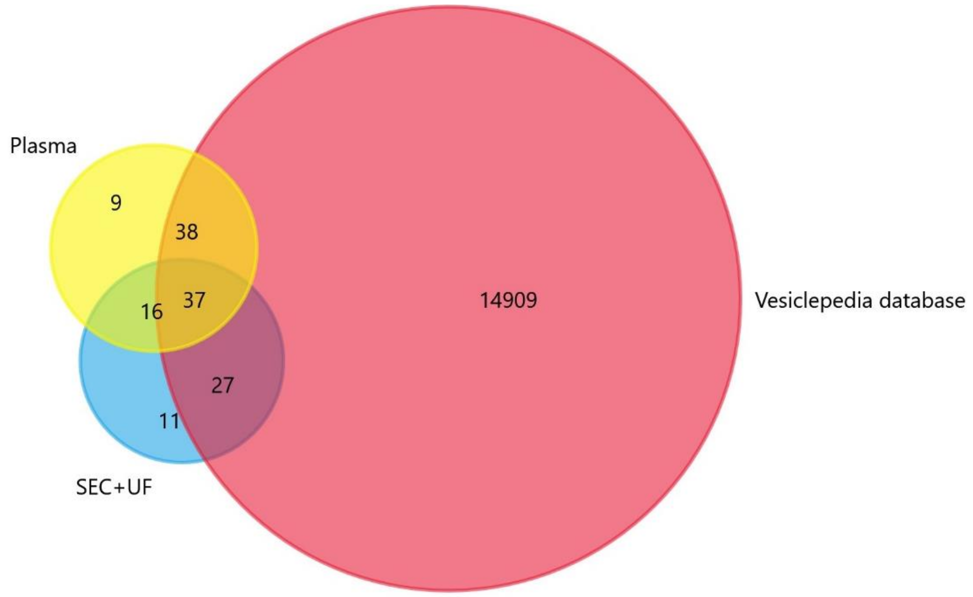

D

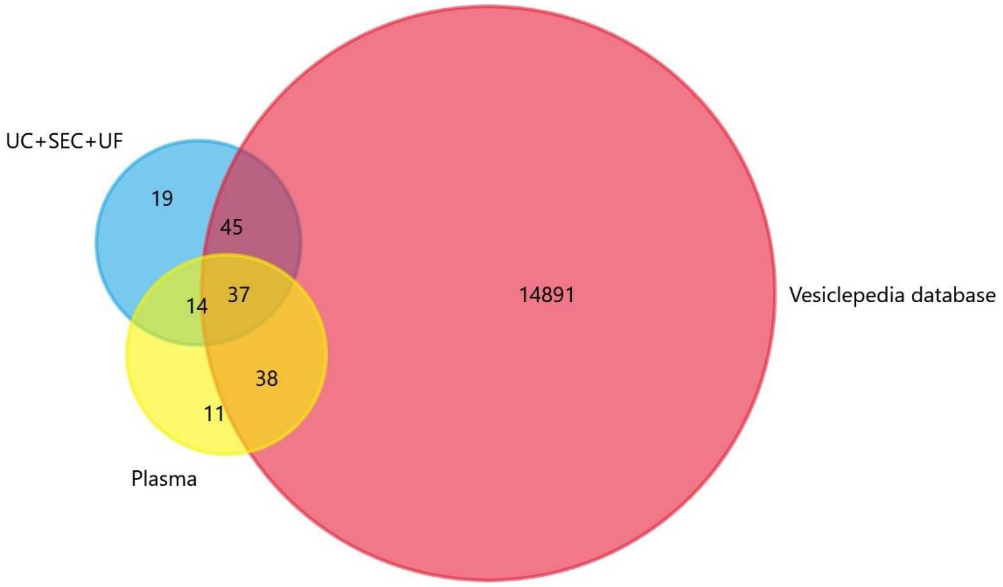

Figure S12 (A-D): Venn diagrams of shared and unique proteins of each sEV enrichment method compared with the complete Vesiclepedia protein database and plasma control.

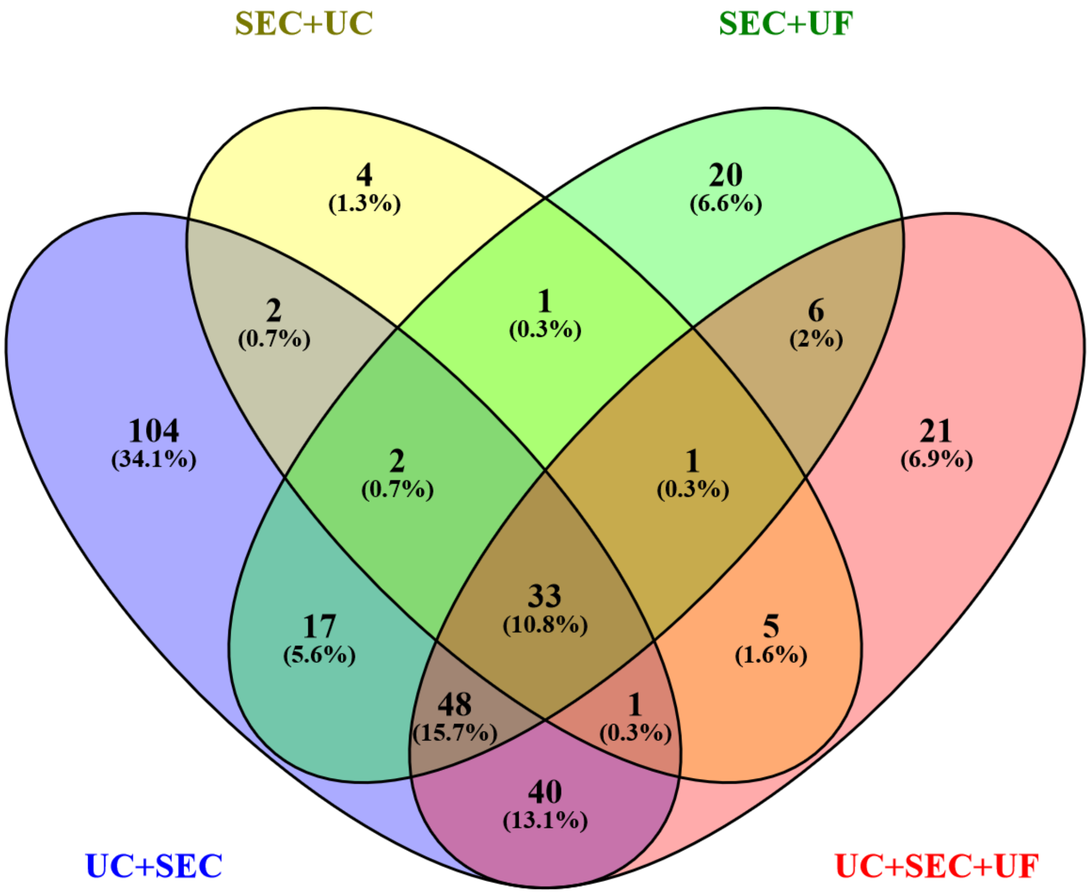

Figure S13: Venn diagram of shared and unique proteins for each method under study identified at 1% FDR protein level and 5% FDR peptide level.

Table S1: Complete list of top 100 EV proteins (ExoCarta) identified in sEV samples and plasma.

ExoCarta EV Protein	UC+SEC	UC+SEC+UF	SEC+UF	SEC+UC	Plasma
CD9	1	1	1	0	0
HSPA8	1	0	0	0	0
GAPDH	1	0	0	0	0
ACTB	1	1	0	0	1
SDCBP	1	1	0	0	0
HSP90AA1	0	0	0	0	0
PKM	1	1	0	0	0
LDHA	0	0	0	0	1
EEF1A1	1	0	0	0	0
YWHAZ	1	0	0	0	0
CLTC	1	0	0	0	0
CD81	1	1	0	0	0
ALB	1	1	1	1	1
VCP	1	1	0	0	0
MSN	1	1	0	0	0
CFL1	1	0	0	0	0
ITGB1	0	1	0	0	0
FLNA	1	0	0	0	0
YWHAG	1	0	0	0	0
A2M	1	1	1	1	1
TUBA1B	1	0	0	0	0
LGALS3BP	1	1	1	1	0
HSPA1A	1	0	0	0	0
PRDX2	0	0	0	0	0
ACTN4	1	0	0	0	0
LDHB	1	0	0	0	1
ACTG1	0	0	1	0	1
TFRC	1	1	0	0	0
THBS1	1	1	1	0	0
AHCY	1	0	0	0	0
STOM	1	1	1	0	0
MYH9	1	0	0	0	0
FN1	1	1	1	0	0
Total	28	15	8	3	6

Table S2: Complete list of top 100 EV proteins (Vesiclepedia) identified in sEV samples and plasma.

Vesiclepedia EV Marker	UC+SEC	UC+SEC+UF	SEC+UF	SEC+UC	Plasma
GAPDH	1	0	0	0	0
HSPA8	1	0	0	0	0
ACTB	1	1	0	0	1
CD9	1	1	1	0	0
PKM	1	1	0	0	0
HSP90AA1	0	0	0	0	0
YWHAZ	1	0	0	0	0
YWHAE	0	0	0	0	0
EEF1A1	1	0	0	0	0
PGK1	0	0	0	0	0
CLTC	1	0	0	0	0
SDCBP	1	1	0	0	0
ALB	1	1	1	1	1
VCP	1	1	0	0	0
CFL1	1	0	0	0	0
MSN	1	1	0	0	0
MYH9	1	0	0	0	0
CD81	1	1	0	0	0
LDHB	1	0	0	0	0
ITGB1	0	1	0	0	0
LDHA	0	0	0	0	1
YWHAG	1	0	0	0	0
ACTN4	1	0	0	0	0
RAB5C	0	0	0	0	0
C3	1	1	1	1	1
FN1	1	1	1	0	0
AHCY	1	0	0	0	0
A2M	1	1	1	1	1
ACTN1	1	0	0	0	0
FLNA	1	0	0	0	0
GSN	1	0	0	0	0
PRDX2	0	0	0	0	0
LGALS3BP	1	1	1	1	0
KRT10	0	0	0	0	1
CAP1	1	1	0	0	0
TFRC	1	1	0	0	0
TLN1	1	0	0	0	0
TUBA4A	1	1	0	0	0
TUBB1	1	0	0	0	0
Total	31	16	6	4	6

Table S3: Number of peptides identified for abundant plasma proteins in sEV enriched samples from the four enrichment methods and plasma control.

	Number of peptide IDs				
**Commonly identified plasma proteins**	**UC+SEC**	**UC+SEC+UF**	**SEC+UF**	**SEC+UC**	**Plasma**
Albumin	61	61	120	94	391
α1-Antitrypsin	8	5	10	2	26
IgA/IgM	25	25	23	6	4
Transferrin	0	25	0	0	129
Haptoglobin	10	11	0	0	3
α2-Macroglobulin	200	137	245	29	164
Fibrinogen	91	69	159	17	49
Complement C3	71	55	76	14	148
α1-Acid Glycoprotein (Orosomucoid)	0	0	0	0	14
HDL (Apolipoprotein A-I)	28	23	37	14	51
HDL (Apolipoprotein A-II)	2	2	2	0	4
LDL (mainly Apolipoprotein B)	11	5	74	0	38
Haemoglobin subunit alpha	7	6	0	0	8
Haemoglobin subunit beta	15	12	9	2	14

Table S4: GO pathway analysis of proteins identified in each sEV-enrichment method. Values represent percentage of total protein IDs. Green = increased in sEV methods and not in plasma. Orange = increased in sEV methods and present in plasma. Red = associated with plasma proteins.

GO pathway	UC+SEC	UC+SEC+UF	SEC+UF	SEC+UC	Plasma Control
Alzheimer disease-presenilin pathway	2.4	5.3	3.6	0	0
Angiogenesis	0	0	0	0	6.5
Angiotensin II-stimulated signaling through G proteins and beta-arrestin	0	0	0	0	3.2
Blood coagulation	24.4	21.1	28.6	44.4	38.7
Cadherin signaling pathway	9.8	10.5	3.6	0	0
CCKR signaling map	7.3	15.8	10.7	0	6.5
Cytoskeletal regulation by Rho GTPase	2.4	0	0	0	0
Huntington disease	2.4	0	3.6	0	0
Integrin signalling pathway	14.6	10.5	7.1	11.1	6.5
p53 pathway	4.9	5.3	3.6	0	0
Parkinson disease	0	0	3.6	0	3.2
Ras Pathway	0	0	7.1	0	6.5
T cell activation	0	0	0	0	6.5
Wnt signaling pathway	9.8	10.5	3.6	0	0

Table S5: List of commonly identified proteins and gene ontology from all methods of sEV enrichment.

Uniprot Accession ID	Gene Name/Gene Symbol/Ortholog	PANTHER Family/Subfamily	PANTHER Protein Class
A0A140T897	Albumin;ALB;ortholog	ALBUMIN (PTHR11385:SF15)	transfer/carrier protein(PC00219)
A0A3Q1LPG0	Uncharacterized protein;unassigned;ortholog	IMMUNOGLOBULIN HEAVY CONSTANT GAMMA 1-RELATED (PTHR23411:SF39)	immunoglobulin receptor superfamily(PC00124)
A0A3Q1M3L6	Uncharacterized protein;unassigned;ortholog	IMMUNOGLOBULIN HEAVY CONSTANT GAMMA 1-RELATED (PTHR23411:SF39)	immunoglobulin receptor superfamily(PC00124)
A0A3Q1M564	Adiponectin;ADIPOQ;ortholog	ADIPONECTIN (PTHR15427:SF20)	scaffold/adaptor protein(PC00226)
A6QNW7	CD5 molecule like;CD5L;ortholog	CD5 ANTIGEN-LIKE (PTHR48071:SF8)	
A7E3W2	Galectin-3-binding protein;LGALS3BP;ortholog	GALECTIN-3-BINDING PROTEIN (PTHR24410:SF16)	defense/immunity protein(PC00090)
F1MLW8	Uncharacterized protein;LOC100847119;ortholog	IG-LIKE DOMAIN-CONTAINING PROTEIN (PTHR23267:SF493)	immunoglobulin(PC00123)
F1N3Q7	Apolipoprotein A-IV;APOA4;ortholog	APOLIPOPROTEIN A-IV (PTHR18976:SF1)	apolipoprotein(PC00052)
F6QND5	Fibrinogen alpha chain;FGA;ortholog	FIBRINOGEN ALPHA CHAIN (PTHR47221:SF3)	
G3N0S9	Uncharacterized protein;unassigned;ortholog		
G3N0V0	Uncharacterized protein;unassigned;ortholog	IMMUNOGLOBULIN HEAVY CONSTANT GAMMA 1-RELATED (PTHR23411:SF39)	immunoglobulin receptor superfamily(PC00124)
G5E513	Uncharacterized protein;unassigned;ortholog	IMMUNOGLOBULIN HEAVY CONSTANT MU (PTHR23411:SF35)	immunoglobulin receptor superfamily(PC00124)
G5E5T5	Uncharacterized protein;unassigned;ortholog	IG-LIKE DOMAIN-CONTAINING PROTEIN (PTHR19944:SF98)	major histocompatibility complex protein(PC00149)
O02659	Mannose-binding protein C;MBL;ortholog	MANNOSE-BINDING PROTEIN C (PTHR24024:SF34)	surfactant(PC00212)
P00974	Pancreatic trypsin inhibitor;unassigned;ortholog	SPLEEN TRYPSIN INHIBITOR I (PTHR10083:SF367)	protease inhibitor(PC00191)
P02070	Hemoglobin subunit beta;HBB;ortholog	HEMOGLOBIN SUBUNIT BETA (PTHR11442:SF42)	globin(PC00107)

P12763	Alpha-2-HS-glycoprotein;AHSG;ortholog	ALPHA-2-HS-GLYCOPROTEIN (PTHR13814:SF6)	protease inhibitor(PC00191)
P15497	Apolipoprotein A-I;APOA1;ortholog	APOLIPOPROTEIN A-I (PTHR18976:SF11)	apolipoprotein(PC00052)
P23805	Conglutinin;CGN1;ortholog	COLLAGEN ALPHA-1(X) CHAIN (PTHR24023:SF880)	extracellular matrix structural protein(PC00103)
P34955	Alpha-1-antiproteinase;SERPINA1;ortholog	ALPHA-1-ANTITRYPSIN (PTHR11461:SF165)	protease inhibitor(PC00191)
Q2KIV9	Complement C1q subcomponent subunit B;C1QB;ortholog	COMPLEMENT C1Q SUBCOMPONENT SUBUNIT B (PTHR15427:SF18)	scaffold/adaptor protein(PC00226)
Q2UVX4	Complement C3;C3;ortholog	COMPLEMENT C3 (PTHR11412:SF81)	protease inhibitor(PC00191)
Q3SYR8	Immunoglobulin J chain;JCHAIN;ortholog	IMMUNOGLOBULIN J CHAIN (PTHR10070:SF2)	immunoglobulin(PC00123)
Q5E9E3	Complement C1q subcomponent subunit A;C1QA;ortholog	COMPLEMENT C1Q SUBCOMPONENT SUBUNIT A (PTHR15427:SF26)	scaffold/adaptor protein(PC00226)
Q7SIH1	Alpha-2-macroglobulin;A2M;ortholog	ALPHA-2-MACROGLOBULIN (PTHR11412:SF165)	protease inhibitor(PC00191)
A5D9E9	Complement subcomponent C1r	Not mapped in PANTHER	
G3MXB5	Uncharacterized protein	Not mapped in PANTHER	
F1N5M2	Gc-globulin	Not mapped in PANTHER	
A0A3Q1LPF0	Apolipoprotein E	Not mapped in PANTHER	
G3MWT1	Ig-like domain-containing protein	Not mapped in PANTHER	
A0A3Q1M032	Uncharacterized protein	Not mapped in PANTHER	
F1MZ96	Uncharacterized protein	Not mapped in PANTHER	
A0A3Q1MNN6	Uncharacterized protein	Not mapped in PANTHER	

Production of StageTips.

Two Empore SCX membrane disks were placed on top of each other and on top of a paper disk in a clean Petri dish. A blunt-end 18g needle was attached to a 1 mL syringe and filled with air. The needle was pressed firmly into the membrane and rotated clockwise to cut out the disks. The syringe and needle containing the double SCX membrane material was inserted into a 300 uL white robotic tip (Eppendorf, cat no: 0030 014.464) and the plunger depressed rapidly down to create positive pressure and eject the membrane into the pipette tip. This was repeated, if necessary, until the double membrane was sitting firmly at the end of the pipette tip.
